# Hypoxia-induced DTL promotes the proliferation, metastasis, and sorafenib resistance of hepatocellular carcinoma through ubiquitin-mediated degradation of SLTM and subsequent Notch pathway activation

**DOI:** 10.1038/s41419-024-07089-4

**Published:** 2024-10-09

**Authors:** Zi-Xiong Chen, Mao-Yuan Mu, Guang Yang, Han Qi, Xiao-Bo Fu, Gui-Song Wang, Wei-Wei Jiang, Bi-Jun Huang, Fei Gao

**Affiliations:** 1 Department of Minimally Invasive Interventional Therapy, Sun Yat-sen University Cancer Center, Guangzhou, 510060 P. R. China; 2 State Key Laboratory of Oncology in South China, Guangdong Provincial Clinical Research Center for Cancer, Sun Yat-sen University Cancer Center, Guangzhou, 510060 P. R. China

**Keywords:** Oncogenes, Liver cancer, Metastasis

## Abstract

Denticleless E3 ubiquitin protein ligase homolog (DTL), the substrate receptor of the CRL4A complex, plays a central role in genome stability. Even though the oncogenic function of DTL has been investigated in several cancers, its specific role in hepatocellular carcinoma (HCC) still needs further elucidation. Data from a clinical cohort (*n* = 209), RNA-sequencing, and public database (TCGA and GEO) were analyzed, indicating that DTL is closely related to patient prognosis and could serve as a promising prognostic indicator in HCC. Functionally, DTL promoted the proliferation, metastasis, and sorafenib resistance of HCC in vitro. In the orthotopic tumor transplantation and tail vein injection model, DTL promoted the growth and metastasis of HCC in vivo. Mechanically, we revealed for the first time that DTL was transcriptionally activated by hypoxia-inducible factor 1α (HIF-1α) under hypoxia and functioned as a downstream effector molecule of HIF-1α. DTL promotes the ubiquitination of SAFB-like transcription modulator (SLTM) and subsequently relieves the transcriptional repression of Notch1. These results suggested that DTL may be a potential biomarker and therapeutic target for HCC.

## Introduction

With the fastest-increasing mortality for decades, liver cancer ranks as the third largest cancer-related death worldwide [1, 2]. Hepatocellular carcinoma (HCC) accounts for ~90% of primary liver cancer, with a 5-year relative survival rate of ~18% [2]. Moreover, two-thirds of HCC patients are diagnosed at advanced stages with limited treatment options and worse prognosis [3]. Whilst our understanding of the molecular pathogenesis of HCC has been improved, few effective molecular targets or biomarkers have been translated into clinical practice [4]. Therefore, there is an urgent need to further understand the mechanisms underlying the HCC progression.

Hypoxia, as the primary characteristic of the tumor microenvironment, plays a vital role in the progression of HCC [5, 6]. As a hypermetabolic tumor, the uninhibited proliferation and abnormal formation of micro-vessels make HCC more susceptible to hypoxia [6]. Hypoxia-inducible transcription factor 1α (HIF-1α), the primary regulator of cellular adaptive responses to hypoxia, extensively participates in the malignant progression and resistance to chemotherapy and immunotherapy of HCC [7, 8]. In the absence of oxygen, HIF-1α binds to hypoxia-responsive elements (HREs) and enhances transcription of downstream target genes [9]. The representative cancer-related genes regulated by HIF-1α include the vascular endothelial growth factor, transforming growth factor (TGF-β), and P53 [10].

Cullin-RING ligases (CRLs), the largest family of E3 ubiquitin ligases, are essential for many eukaryotic biological processes, including cell-cycle progression and maintenance of genomic stability [11]. Denticleless E3 ubiquitin protein ligase homolog (DTL), also known as CDT2, DCAF2, or RAMP, serves as the substrate receptor of the CRL4^Cdt2^ ubiquitin ligase complex [12]. The unique substrate recognition paradigm of CRL4^Cdt2^ depends on the prior interaction of its substrates with chromatin-bound PCNA [13]. DTL is responsible for recognizing multiple substrates such as Cdt1, E2F1, Set8, and p21 [13–16]. To date, the role of CRL4^Cdt2^ in maintaining genome integrity has been well clarified [13, 17]. Due to the central part of DTL in genome stability and its multiple substrates, dysregulation of DTL is associated with tumorigenesis [18, 19]. Previous studies have shown that overexpression of DTL correlated with adverse outcomes in certain tumors, such as cervical cancer, gastric cancer, and melanoma [20–22]. However, the underlying molecular mechanism for DTL dysregulation and the role of DTL in HCC malignant progression remain unclear.

The SLTM [scaffold attachment factor (SAF) B-like transcription modulator], a member of the SAF-like transcriptional modulators, features a DNA-binding SAF-box motif and an RNA-binding domain [23]. SLTM is localized in the nucleus and known for its role in transcription repression. Knockdown of SLTM results in dysregulation of cell-cycle progression, chromatin condensation and cytochrome *c* release, consequently triggering programmed cell death [24]. However, the potential relationship between DTL and SLTM and its subsequent impact on the progression of HCC requires further investigation.

In this study, the biological significance of DTL was verified based on a retrospective patient cohort, HCC RNA-sequencing (RNA-seq) data, and public databases (The Cancer Genome Atlas, TCGA and Gene Expression Omnibus, GEO). Functionally, DTL promoted HCC proliferation, metastasis, and sorafenib resistance. Mechanistically, we demonstrated hypoxia-induced DTL expression through a HIF-1α-dependent manner for the first time. Elevated DTL expression activated epithelial–mesenchymal transition (EMT) through the Notch pathway. In general, this study suggested DTL as a promising biomarker of HCC malignant progression. It revealed a novel HIF-1α/DTL/SLTM signaling pathway that may serve as a potential target for HCC therapy.

## Results

### DTL is upregulated in HCC tissues and associated with poor prognosis

**Fig. 1:**
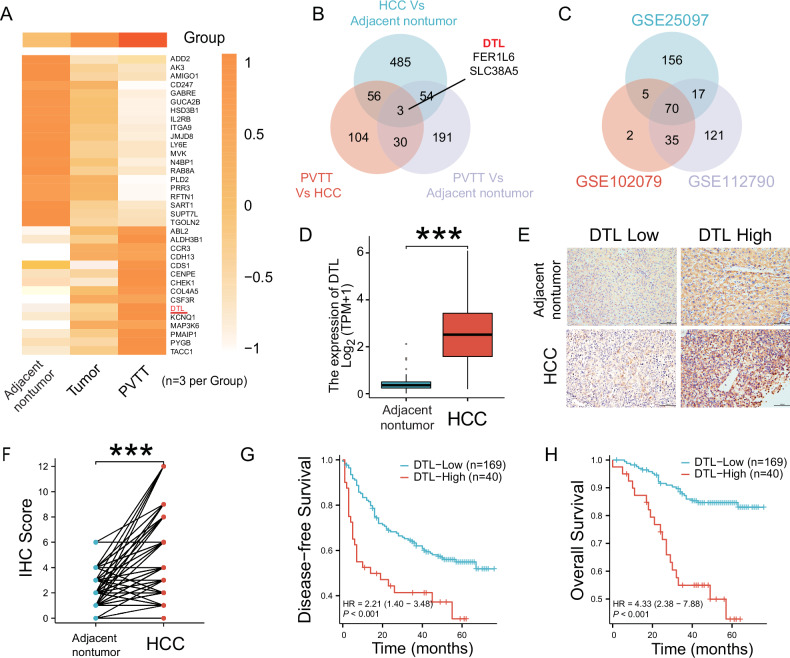
DTL is overexpressed in human HCCs and correlates with poor prognosis. **A** Heatmap depicting mRNA sequencing data from nine samples, which include three pairs of PVTT, tumor, and adjacent non-tumor tissues. The Venn diagrams demonstrate the overlap of differentially expressed genes (DEGs) across these samples (**B**) and three HCC datasets from GSE databases (**C**). **D** DTL transcriptional expression in patients with HCC and adjacent non-tumor from profiling data on TCGA databases. **E** Representative IHC staining showed the DTL expression level in HCC and adjacent non-tumor tissues (×100). **F** IHC score analysis of DTL in HCC and adjacent non-tumor samples (*n* = 209). **G**, **H** Disease-free survival and overall survival in DTL-high and DTL-low groups. ***P < 0.001.

RNA-seq was performed on nine tissue samples, consisting of three matched sets of non-tumor, tumor, and portal vein tumor thrombosis (PVTT) tissues from three patients. The results indicated that DTL expression levels increased successively in adjacent non-tumor, HCC, and PVTT tissue (Fig. 1A, B and Supplementary Fig. 1B–D). To further verify this finding, datasets GSE112790, GSE25097, and GSE102079 were retrieved from the GEO database. After normalizing the expression profile, upregulated expression of DTL was observed in HCC tissues compared with adjacent normal liver tissues in the three datasets (Fig. 1C and Supplementary Fig. 1A). Similarly, the expression level of DTL in the HCC dataset from the TCGA database was also higher than in normal liver tissue (unpaired or paired) (Fig. 1D and Supplementary Fig. 2A); HCC patients with higher DTL expression level correlated with shorter overall survival (OS) based on TCGA database (Supplementary Fig. 2B). In addition, DTL was higher in advanced pathological grade, T and N stages (Supplementary Fig. 2C–E). The area under the curve (AUC) under the receiver operating characteristics (ROC) curve was 0.926, indicating that DTL had excellent predictive ability for HCC (Supplementary Fig. 2F). Therefore, DTL was selected for further experiments.

To explore whether DTL expression was associated with HCC patients’ clinical features and prognosis, we retrospectively collected clinical information and tissue samples after obtaining approval from the Ethics Committee of Sun Yat-sen University Cancer Center. A total of 209 patients who underwent surgical resection and were pathologically confirmed as HCC from January 2017 to January 2019 were included in this study. Immunohistochemistry (IHC) staining was performed on paraffin-embedded tissue sections. DTL expression level in HCC tissue was significantly higher than in adjacent non-tumor tissue (*P* < 0.001; Fig. 1E, F).

According to the cutoff values determined by the X-tile, patients were divided into two groups: DTL-high expression (IHC ≥ 6, *n* = 40) and DTL-low expression group (IHC < 6, *n* = 169). Supplementary Table 1 shows the baseline characteristics of patients in both groups. The upregulated expression of DTL was significantly associated with vascular invasion (*P* < 0.001) and advanced TNM stage (*P* < 0.001) (Supplementary Table. 1). The results of KM survival analysis suggest that patients with high expression of DTL exhibited a poorer OS (*P* ≤ 0.001) or disease-free survival (DFS) (*P* ≤ 0.001) (Fig. 1G, H). Moreover, the univariate analysis showed that tumor differentiation, tumor size, vascular invasion, TNM stage, and DTL level were significantly associated with OS and DFS in HCC patients, while age stage was associated only with OS (*P* < 0.05); Multivariate analysis demonstrated that tumor size (>3.0 cm) and high DTL level were independent risk factors for both OS and DFS (*P* < 0.05) (Supplementary Tables 2 and 3). These results indicated that the upregulation of DTL is significantly related to HCC progression and poor clinical prognosis.

### DTL is induced by hypoxia

**Fig. 2:**
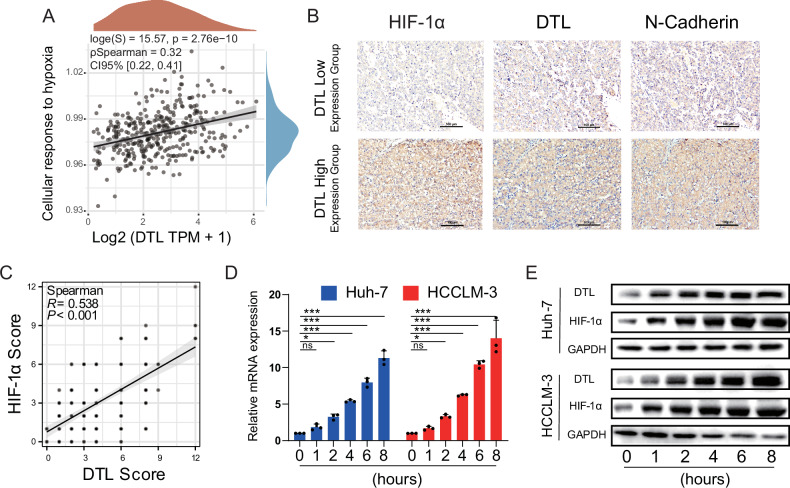
DTL expression was positively correlated with hypoxia. **A** The correlation between DTL and cellular response to the hypoxia pathway was analyzed with Spearman. **B** Representative IHC staining of HIF-1α, DTL, and *N*-cadherin in DTL-high and DTL-low HCC tissue. **C** Correlation analysis between the IHC score of DTL and HIF-1α (*n* = 209). **D** The mRNA levels of DTL at different hypoxia time points. **E** Protein expression of DTL and HIF-1α at different time points. *P < 0.05, ***P < 0.001, ns no significance.

To find the underlying mechanism of the upregulated expression of DTL in HCC, the TCGA-liver cancer database was used to explore the correlation between DTL and multiple pathways with Spearman correlation analysis. The results indicated that DTL was closely related to the hypoxia-related pathway (Fig. 2A) [25], DNA damage repair-related pathways (DNA-repair), and proliferation-related pathways (proliferation-signature, G2M-checkpoint, DNA-replication) (Supplementary Fig. 3). Previous studies have suggested that DTL induces proliferation, metastasis, and invasion of cancer cells through several different signaling pathways [19, 22, 26]. As a common feature of solid tumors, the hypoxia microenvironment mediates expression changes in many downstream genes [6]. Therefore, we investigated whether the hypoxia tumor microenvironment is involved in the upregulated expression of DTL. IHC staining and scoring of HIF-1α and DTL were performed in 209 HCC samples (Fig. 2B). We found that DTL expression, as expected, positively correlated with HIF-1α (*R*^2^ = 0.409, *P* < 0.001) (Fig. 2C). Then, Huh-7 and HCCLM-3 cells were cultured in hypoxic atmosphere (1% O_2_). qRT-PCR and western blotting were used to detect the expression of HIF-1α and DTL at time points 0, 1, 2, 4, 6, and 8 h. The results showed that DTL mRNA/protein levels increase in response to hypoxia (Fig. 2D, E). The results described above indicated that abnormal expression of DTL may be caused by hypoxia.

### HIF-1α activates DTL expression by binding to HRE in the DTL promoter region

HIF-1α, a key transcription factor in the microenvironment, induces the expression of its downstream target genes and promotes HCC proliferation, angiogenesis, and metastasis [6, 8, 27]. Therefore, we hypothesize that HIF-1α transcriptionally upregulates the expression of DTL under hypoxia conditions.

**Fig. 3:**
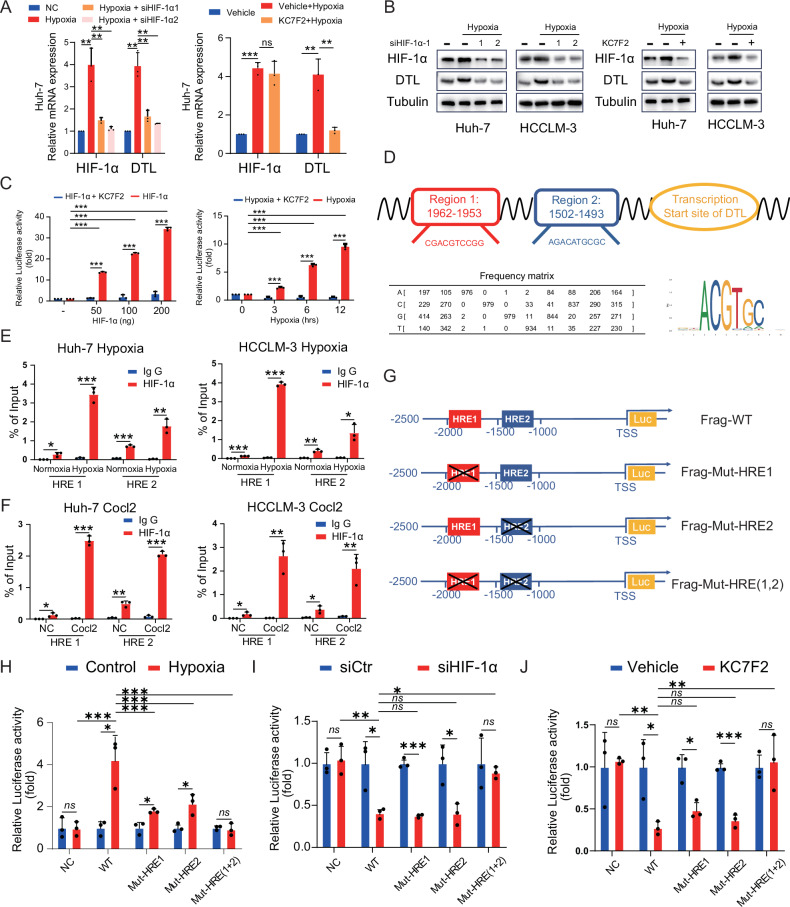
HIF-1α activates DTL transcription under hypoxia. **A**, **B** HCC cells were transfected with HIF-1α-siRNA or treated with KC7F2 (40 μM) under hypoxia (1% O_2_). The mRNA and protein expression of DTL and HIF-1α were determined using qRT-PCR and a western blot assay. **C** Dual-luciferase reporter assays were performed in HEK-293T cells with HIF-1α overexpression or hypoxia following KC7F2 treatment (40 μM). **D** Sequences of the predicted HRE in the DTL promoter. **E**, **F** Chromatin immunoprecipitation (ChIP) assays in Huh-7 and HCCLM-3 cells to identify the binding of HIF-1α to the DTL promoter under hypoxia or after treatment with CoCl_2_. **G** Schematic diagram of the DTL promoter and three mutant constructs. **H** Dual-luciferase reporter assays for the mutant HRE sequences in HEK-293T cells under hypoxia. **I**, **J** Dual-luciferase reporter assays for the mutant HRE sequences in HEK-293T cells following siHIF-1α and KC7F2 (40 μM) under hypoxia. *P < 0.05, **P < 0.01, ***P < 0.001, ns no significance.

After inhibiting HIF-1α with small interfering RNA (siRNA), the expression of HIF-1α and DTL significantly decreased at both mRNA and protein levels (*P* < 0.01) (Fig. 3A, B and Supplementary Fig. 4A). The small-molecule inhibitor KC7F2A, which selectively inhibits HIF-1α mRNA translation, resulted in a significant decrease of HIF-1α and DTL at the protein level (Fig. 3B and Supplementary Fig. 4B). Dual-luciferase reporter assay employed under HIF-1α overexpression or hypoxia conditions, demonstrating that HIF-1α binds to the DTL promoter and activates its transcription (*P* < 0.001); the use of KC7F2A significantly inhibited luciferase intensity indicating that DTL expression is mainly regulated by HIF-1α under hypoxic conditions (Fig. 3C). JASPAR transcription factor database was used to detect potential HRE within the −2000 to +50 bp region of DTL transcription start site. We observed two putative HRE within the genomic region (Fig. 3D). Then, chromatin immunoprecipitation (ChIP) using an antibody to HIF-1α and qRT-PCR was performed on the putative HRE under hypoxia (1% O_2_) or cobalt chloride (CoCl_2_, 200 μM) conditions. These results suggest that HIF-1α could directly bind to both HRE1 and HRE2 of the DTL promoter region in Huh-7 and HCCLM-3 cells (Fig. 3E, F). Next, we constructed an HRE mutated vector in turn, as shown in Fig. 3G. Luciferase activity was measured under the conditions of hypoxia, hypoxia + siHIF-1α, and hypoxia + KC7F2; mutating HRE1 or HRE2 respectively resulted in a significant decrease in luminescence intensity under hypoxic conditions. Additionally, HRE (1 + 2) mutation restored luciferase activity under hypoxic to the normoxic conditions level (Fig. 3H). Similarly, siHIF-1α or KC7F2 can significantly reduce the luciferase activity of HEK-293T cells under hypoxic conditions, and the luciferase activity of HRE1 or HRE2 mutant group decreased significantly. Moreover, the luciferase activity of HRE1 + 2 mutant group showed no significant changes after siHIF-1α or KC7F2 treatment (Fig. 3I, J). In light of these results, we can conclude that DTL is a direct transcriptional target of HIF-1α, and HRE1 and 2 are crucial in this process.

### DTL promotes HCC proliferation and sorafenib resistance

**Fig. 4:**
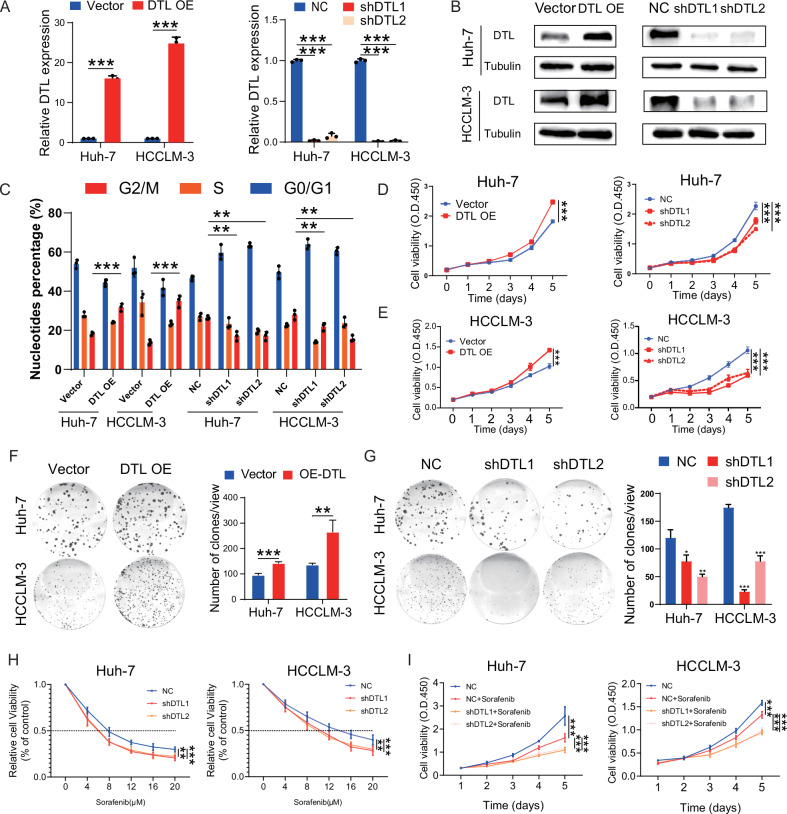
DTL promotes cell-cycle progression, proliferation, and sorafenib resistance in HCC cells. **A**, **B** DTL overexpression and knockdown stable cell line were established; the DTL mRNA and protein levels were determined by qRT-PCR and western blot. **C** The effect of DTL overexpression and knockdown on the cell cycle in Huh-7 and HCCLM-3 were analyzed by flow cytometry. **D**, **E** The effect of DTL overexpression and knockdown on proliferation in Huh-7 and HCCLM-3 cells was determined by Cell Counting Kit-8 (CCK-8) assays. **F**, **G** The effect of DTL overexpression and knockdown on proliferation in Huh-7 and HCCLM-3 cells was determined by clone formation assay. **H** The relative cell viability of DTL knockdown Huh-7 and HCCLM-3 cells after treatment with different concentrations of sorafenib for 72 h was detected by CCK-8 assays. **I** CCK-8 assays showed the proliferation of Huh-7 and HCCLM-3 cells response to 3 μM sorafenib with DTL knockdown. *P < 0.05, **P < 0.01, ***P < 0.001, ns no significance.

To investigate the oncogenic function of DTL in HCC cells, lentivirus-mediated stable overexpression and knockdown of DTL cell lines were constructed. The DTL knockdown cell lines were constructed with a doxycycline-inducible system since the poor growth status was caused by DTL silence. DTL knockdown cell lines were cultured in Dulbecco’s modified Eagle’s medium (DMEM) containing 10% tetracycline-free serum (ABWBIO, China). The knockdown effect was satisfactory after 48 h of doxycycline induction (1 μg/mL) (Supplementary Fig. 4C). Stable cell lines were verified through qPCR and WB analysis (Fig. 4A, B). For all subsequent in vitro experiments, DTL-shRNA cell lines were conducted after 48 h of doxycycline induction (1 μg/mL). Cell-cycle analysis by flow cytometry was performed to test changes in the Huh-7 and HCCLM-3 cell lines after DTL overexpression and knockdown. Overexpression of DTL promotes cell cycle through increasing the G2/M phase population, and knockdown of DTL induces G2/M cell-cycle arrest (Fig. 4C and Supplementary Fig. 4D). Cell proliferation ability was detected by CCK-8 proliferation assay and colony formation assay. These results suggested that DTL promoted the proliferation of HCC. In turn, silencing of DTL severely slowed growth rates and impaired the colony formation abilities of HCC cells (Fig. 4D–G).

Sorafenib, a multi-kinase inhibitor, is the standard therapy for advanced HCC with limited survival benefit [28]. Multiple genes involved in the proliferation, migration, or invasion of HCC were related to sorafenib resistance [29]. In this study, we found the knockdown of DTL-sensitized Huh-7 and HCCLM-3 cells’ response toward sorafenib treatment (Fig. 4H). Similarly, the Cell Counting Kit-8 (CCK-8) assays also indicated that DTL knockdown combined with sorafenib exhibited a more substantial inhibitory effect on HCC cells (Fig. 4I).

### DTL promotes HCC invasion and metastasis in vivo and in vitro

**Fig. 5:**
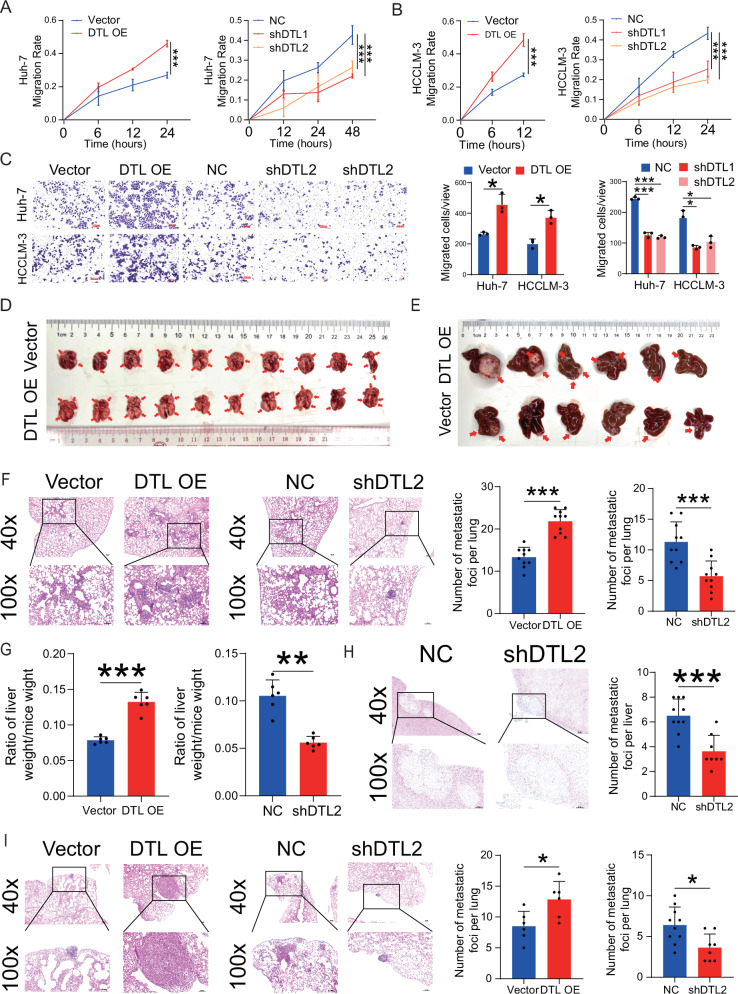
DTL promoted metastasis of HCC cells in vitro and vivo. **A**, **B** The effect of DTL overexpression and knockdown on Huh-7 and HCCLM-3 cells wound healing. **C** Transwell assays were performed to evaluate the migration ability of DTL overexpression and knockdown HCC cells. **D** Representative images of lung tissues in tail vein tumor metastasis mouse model from DTL overexpression and vector groups. **E** Representative images of liver tissues in orthotopic liver transplantation model from DTL overexpression and vector groups. **F** Representative images of lung metastatic nodules in hematoxylin–eosin (HE) stained sections. The left panel shows the number of lung nodules in DTL overexpression and vector groups from the tail vein tumor metastasis mouse model. **G** The relative liver weight was calculated as the ratio of wet liver weight to body weight ratio. **H** Representative images of intrahepatic metastasis nodules in HE‐stained sections. Left panel: the number of intrahepatic metastasis nodules in the DTL knockdown group. **I** Representative images of lung metastatic nodules in HE‐stained sections. Left panel: the number of lung nodules in DTL overexpression and vector groups from orthotopic liver transplantation model. *P < 0.05, **P < 0.01, ***P < 0.001, ns no significance.

To explore the effect of DTL on HCC cells migration and invasion ability, we performed in vitro wound-healing and transwell assays. DTL overexpression significantly enhanced HCC cells migration ability and increased the number of cells migrating/invading through the membrane compared to the empty vector. By contrast, DTL knockdown markedly suppressed the mobility and invasiveness of Huh-7 and HCCLM-3 cells (Fig. 5A–C and Supplementary Fig. 5A, B). Moreover, to detect the effect of DTL on the proliferation and metastasis of HCC in vivo, we contrasted the tail vein metastasis model and orthotopic HCC implantation model with HCCLM-3 DTL-overexpression, knockdown, and their corresponding control cells; paraffin-embedded tissue sections were hematoxylin–eosin (HE) stained to detect the intrahepatic tumor and lung metastatic. The results showed that the number of metastatic lung nodules in the DTL overexpression group (*n* = 10) was significantly increased compared to the control group (*n* = 10); the DTL knockdown group (*n* = 10) displayed the opposite results (Fig. 5D, F and Supplementary Fig. 5C). Likewise, in the liver orthotopic transplantation model, DTL promoted tumor growth and metastasis in terms of wet weight of liver, ratio of liver weight/mice weight and intrahepatic metastatic foci compared with the control group (*n* = 6); in contrast, knockdown of DTL reduced wet weight of liver, liver/mice weight ratio and the number of intrahepatic metastatic nodules (*n* = 10) (Fig. 5E, G, H and Supplementary Fig. 5D–F). The number of lung metastatic nodules in the liver orthotopic transplantation model was also counted and reached similar conclusions (Fig. 5I and Supplementary Fig. 5G, H). These results suggest that DTL enhances HCC cell proliferation, invasion, and metastasis in vivo and in vitro.

### DTL plays a crucial role in hypoxia-induced proliferation and metastasis of HCC

**Fig. 6:**
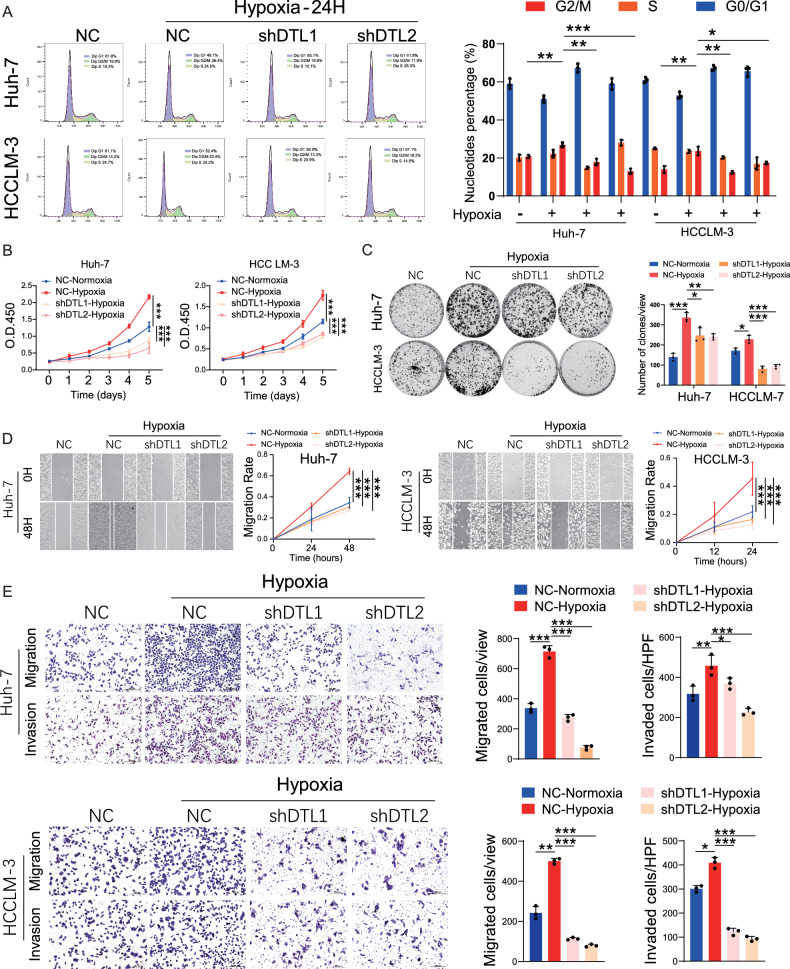
The oncogenic role of HIF-1α in HCC cells partly depended on DTL. **A** Cell-cycle analysis evaluated the effects of DTL knockdown on cell cycle under hypoxia in HCC cells. **B**, **C** Cell Counting Kit-8 (CCK-8) and clone formation assays were performed to evaluate the DTL knockdown on the proliferation of HCC cells under hypoxia. **D** Representative images and quantification of wound-healing assays for DTL knockdown HCC cells under hypoxia. **E** Representative images and quantification of transwell migration and invasion assays for DTL knockdown HCC cells under hypoxia. *P < 0.05, **P < 0.01, ***P < 0.001, ns no significance.

Hypoxia/HIF-1α promotes proliferation and metastasis of HCC cells [7, 30]. As hypoxia increases DTL expression in HCC, we examined whether DTL was required in mediating hypoxia-induced proliferation and metastasis of HCC cells. As depicted in Fig. 6A, the suppression of DTL effectively hindered the hypoxia-induced increase of the G2/M cell-cycle phase in both Huh-7 and HCCLM-3 cells. CCK-8 and colony formation assays showed that silencing DTL suppressed the hypoxia-induced proliferation of HCC cells (Fig. 6B, C). The scratch test and transwell assays indicated that hypoxia-induced cell migration and invasion were attenuated by DTL knockdown (Fig. 6D, E). All the above results indicate that DTL plays a vital role in hypoxia-induced proliferation and metastasis of HCC.

### DTL induces EMT through the Notch pathway

**Fig. 7:**
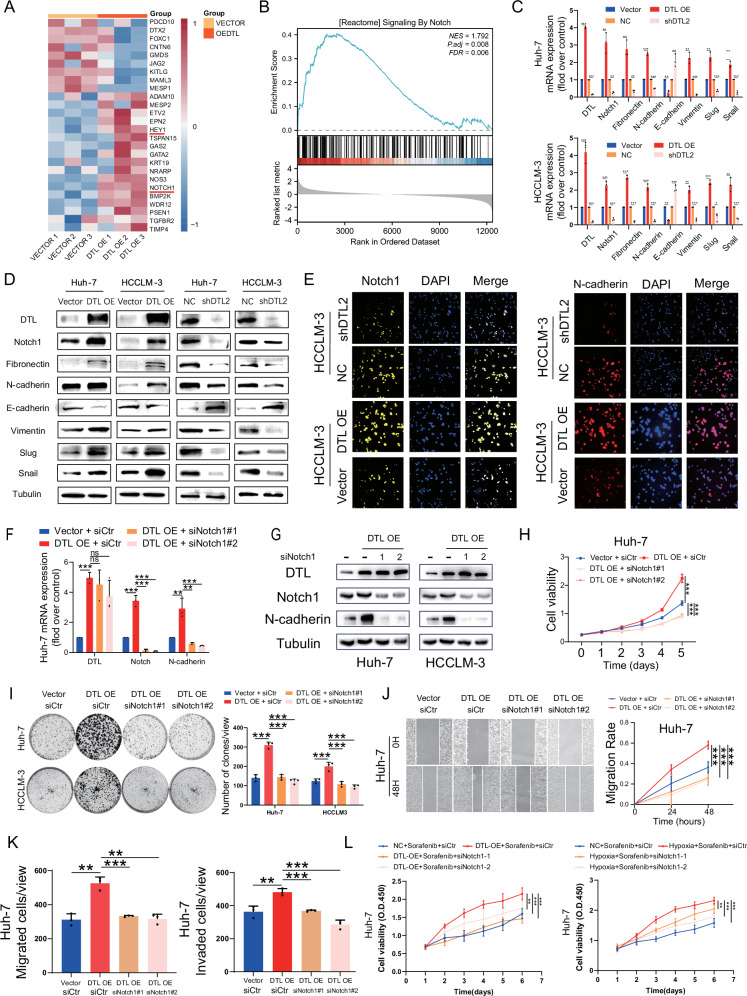
Notch signaling pathway mediated DTL-induced EMT, proliferation, and metastasis of HCC cells. **A** Heatmap of the representative upregulated and downregulated gene after overexpression of DTL in HCCLM-3 cells. **B** Gene set enrichment analysis (GSEA) of the Notch pathway. **C** qRT-PCR results of Notch1 and EMT-related genes mRNA expression in DTL overexpressing or knockdown HCC cells. **D** Western blot results of Notch1 and EMT-related genes protein expression in DTL overexpressing or knockdown HCC cells. **E** Immunofluorescence results of Notch1 and *N*-cadherin expression in DTL overexpressing or knockdown HCCLM-3 cells. **F**, **G** qRT-PCR and western blot detected the knockdown efficiency of Notch1 by siRNA. **H**, **I** The functional role of Notch1 in DTL-induced proliferation of HCC cells was detected by Cell Counting Kit-8 (CCK-8) and clone formation assays. **J**, **K** The functional role of Notch1 in DTL-induced metastasis of Huh-7 cells was detected by wound-healing and transwell assays. **L** CCK-8 assay evaluating Huh-7 cells viability under DTL overexpression or hypoxia with Notch1 knockdown and sorafenib (3 μM) treatment. *P < 0.05, **P < 0.01, ***P < 0.001, ns no significance.

To elucidate the potential molecular mechanisms underlying the DTL in promoting HCC malignant progression, we performed RNA-seq on DTL overexpression HCCLM-3 cells and its control cell line (Fig. 7A). Gene set enrichment analysis (GSEA) results suggest that upregulated DTL may activate the Notch signaling pathway (Fig. 7B). Consistently, the results of qRT-PCR, western blot, and immunofluorescence showed that upregulation of DTL resulted in a significant increase of the Notch1 and EMT-related gene levels, while silencing of DTL had an opposite effect (Fig. 7C–E and Supplementary Fig. 6A). Next, to investigate the impact of Notch pathway on DTL’s oncogenic role in HCC cells, we performed rescue assays with Notch1 small interfering RNA (siNotch1). Both targets on Notch1 siRNA could effectively inhibit the expression of Notch1 at the mRNA and protein level (Fig. 7F, G and Supplementary Fig. 6B). As shown in Fig. 7H, I and Supplementary Fig. 6C, the CCK-8 and colony formation assays demonstrated that siNotch1 significantly inhibited DTL-induced proliferation in HCC cells. On the other hand, results of the scratch test and transwell assays suggested that the migration and invasion capabilities induced by DTL were partly reversed by the siNotch1 (Fig. 7J, K and Supplementary Fig. 6D–F). We further observed that HCC cells displayed increased tolerance to sorafenib with DTL overexpression or under hypoxic conditions, which was significantly reduced upon Notch1 knockdown (Fig. 7L and Supplementary Fig. 6G). Taken together, our results suggest that DTL accelerates HCC cells proliferation, invasion and sorafenib resistance by activating the Notch signaling pathway.

### DTL-mediated ubiquitination and degradation of SLTM

**Fig. 8:**
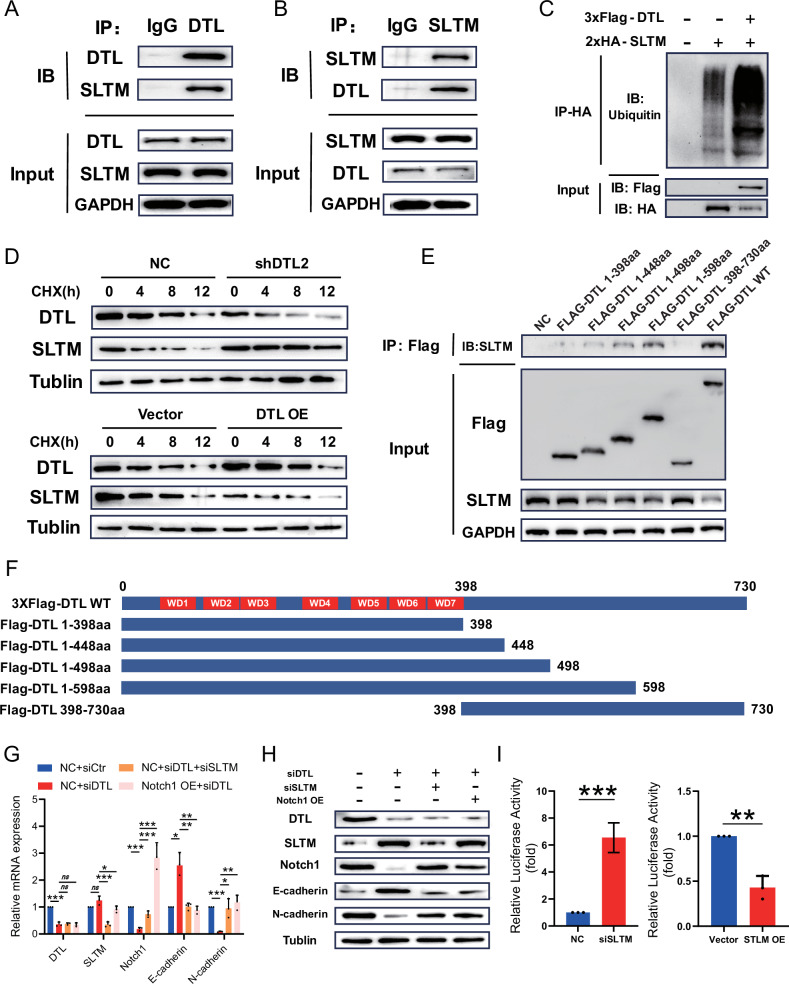
DTL directly binds to SLTM and mediates its ubiquitination-dependent degradation. **A**, **B** Co-immunoprecipitation (Co-IP) and western blot (WB) were performed to assess the interaction between exogenous DTL and SLTM in Huh-7 cells. **C** 2xHA-SLTM was transfected into 293T cells with or without 3xFlag-DTL. The effect of DTL on SLTM ubiquitination was analyzed by IP and WB. **D** WB analysis of 293T cells with DTL knockdown and those overexpressing DTL, both treated with cycloheximide (CHX, 50 mg/ml) for various time points to evaluate protein degradation. **E** Co-IP was employed to determine the binding domain of DTL that interacts with SLTM. **F** Schematic overview of the full-length DTL and its truncated mutants, depicted in plasmid constructs. **G**, **H** Quantitative real-time PCR (qRT-PCR) and WB were conducted to assess the expression levels of cleaved Notch1, as well as the Notch1 pathway downstream target genes, in Huh-7 cells. **I** Luciferase reporter for the Notch1 in 293T cells with SLTM overexpression or knockdown. *P < 0.05, **P < 0.01, ***P < 0.001, ns no significance.

In order to clarify the specific molecular mechanisms of DTL regulating the Notch pathway, interaction proteins of DTL were assessed using co-immunoprecipitation (Co-IP). A Huh-7 cell line stably expressing 3xFlag-tagged DTL was constructed using lentivirus (Supplementary Fig. 7). Cell lysates were subjected to immunoprecipitation (IP) with an anti-FLAG antibody to precipitate the FLAG-tag DTL protein. After washing, the protein of interest was eluted from the resin, followed by tryptic digestion and liquid chromatography–tandem mass spectrometry (LC–MS/MS) analysis. The results drew our attention to SLTM, a protein primarily involved in transcriptional repression, which is exclusive to the experimental group (Supplementary Fig. 7). The interaction between DTL and SLTM was verified by Co-IP assay in the Huh-7 cells with DTL antibody (and vice versa) (Fig. 8A, B). To further evaluate the role of SLTM as a ubiquitination substrate of DTL, we investigated whether DTL could induce the ubiquitination of SLTM. As a result, efficient ubiquitination of SLTM was observed upon the overexpression of DTL in the 293T cells (Fig. 8C). Following treatment with protein synthesis inhibitor cycloheximide (CHX, 50 mg/ml), we observed that overexpression of DTL facilitated the degradation of SLTM, whereas knockdown of DTL markedly inhibited this process (Fig. 8D). Additionally, we constructed a variety of DTL truncation mutants, and Co-IP assays demonstrated that the interaction with SLTM is localized within the aa 498 to 598 region of DTL (Fig. 8E, F).

To verify the correlation between SLTM and the Notch1 pathway, we investigated the capacity of SLTM to modulate the expression of Notch1 and its downstream effectors. Our results indicate that SLTM expression is negatively correlated with that of Notch1 and can modulate the expression of EMT-associated molecules (Fig. 8G, H). Finally, through a dual-luciferase reporter assay, we confirmed that SLTM can regulate the expression of Notch1 by suppressing its transcription (Fig. 8I).

Collectively, these results demonstrate that DTL can bind to and facilitate the ubiquitination of SLTM, leading to its consequent ubiquitin-proteasomal degradation. SLTM exerts regulatory control over the transcription of Notch1, which in turn affects the expression of its downstream effectors.

## Discussion

DTL, a PCNA-dependent E3 ubiquitin ligase, plays a central role in the maintenance of genome integrity. Molecular mechanisms of DTL participating in the physiological cell and DNA damage response have been extensively studied [18]. Given the central role of DTL, recent studies have demonstrated that DTL is highly expressed in various cancers and involved in cancer progression [19, 22, 31]. Previous Studies have reported that DTL functionally promotes the cell cycle and affects immune cell infiltration in HCC [32, 33]. However, the role of DTL in other malignant phenotypes and its underlying molecular mechanisms remain poorly understood. On the other hand, the mechanism triggering the upregulation of DTL remains unclear.

First, to verify the correlation between DTL level and patient prognosis, we recruited the largest HCC cohort to date; data from RNA-seq and public databases were collected and analyzed. The results indicated a significant correlation between high DTL expression and poor prognosis in HCC patients. Furthermore, DTL level showed a significant correlation between higher TNM staging, the absence of vascular invasion, and PVTT, as shown in Supplementary Table 1. Subsequently, the functional role of DTL in promoting proliferation and metastasis of HCC was verified through in vivo and in vitro experiments. Due to the absence of vascular invasion or distant metastasis, more than 50% of patients are diagnosed at an advanced stage, and ~70% of patients suffer from relapse within 5 years [34, 35]. Sorafenib is known as a cornerstone treatment for advanced HCC. However, only about 30% of patients are benefitted from sorafenib, and drug resistance usually develops within 6 months [29]. Enhanced proliferation and cell motility are essential for the development of sorafenib resistance. Many genes that participate in the proliferation or migration of HCC cells can promote resistance to sorafenib [36, 37]. Our results indicated that DTL downregulation improves the sensitivity of HCC cells to sorafenib, which could provide new therapeutic targets for overcoming the drug resistance of sorafenib.

As one of the most striking features of solid tumors, the hypoxia tumor microenvironment contributes to the malignant phenotype and aggressive tumor behaviors [38]. HIF-1α is one of the most commonly oncogenic transcriptional regulators and participates in almost all cellular processes [39]. Upregulation of HIF-1α was reported to promote growth, metastasis, and sorafenib resistance [40, 41]. While inhibition of HIF-1α has long been considered a potential therapeutic target, effective drugs, as well as deeper mechanisms, still need to be investigated [42]. In our study, we revealed a hypoxia-dependent transcriptional activation molecular mechanism of DTL mediated by HIF-1α for the first time. ChIP assays showed that HIF-1α directly binds to the hypoxia response element on the DTL promoter. The upregulation of DTL under hypoxia was mainly mediated by HIF-1α. Since, in the absence of HRE on the DTL promoter, hypoxia could not induce transcriptional activation. Furthermore, the functional importance of DTL in the hypoxia-induced malignant progression of HCC was also confirmed. These results suggest that DTL is a novel functional mediator downstream of hypoxia/ HIF-1α signaling.

EMT is the initial process of tumor cells taken to invade and metastasize [43]. Under hypoxia, multiple signaling pathways were activated, including TGF-β, NF-κB, phosphoinositide 3-kinase (PI3K)/Akt, and Notch1 [44, 45]. As a highly conserved pathway, the notch signaling pathway plays a critical role in the initiation and progression of different cancers [46]. Notch receptor is a heterodimer receptor composed of the Notch extracellular domain, transmembrane, and Notch intracellular domains (NICD). Once interacting with a transmembrane ligand, the NICD is released and translocates into the nucleus. Then, a ternary complex, formed by NICD and the members of the CSL transcription factor family, mediates the transcription of downstream genes [45]. It has been established that the Notch1 pathway can facilitate the invasion, metastasis, and proliferation of liver cancer [47]. Furthermore, existing studies have demonstrated that molecules such as YTHDF1 and HOIL-1 can mediate increased sorafenib resistance in liver cancer through the Notch1 pathway [48, 49].

Pathway analysis of the RNA-seq analysis revealed that DTL expression was positively correlated with the Notch signaling pathway. The expression detection of NICD and several downstream markers indicated that DTL can activate the Notch pathway and facilitate EMT. The DTL-mediated proliferation, metastasis, and EMT of HCC can also be reversed by the siNotch1. The study by Jin et al. indicated that DTL exerted carcinogenic effects by inducing the c-Jun N-terminal kinase (JNK) pathway in cervical adenocarcinoma [22]. Our sequencing results showed that DTL resulted in a more obvious activation of the Notch1 pathway, which indicated the underlying oncogenic mechanisms of DTL were not the same. However, the mechanism underlying the interaction between DTL and the Notch1 signaling pathway remains unclear.

Hence, we performed LC–MS/MS to explore proteins that might interact with DTL and potentially modulate the Notch1 pathway. Among the proteins specifically identified in the experimental group, we discovered that the SLTM is consistent with our hypotheses. Subsequent experiments have also confirmed its interaction with DTL and its regulatory influence on the Notch1 pathway. SLTM, a member of the SAFB family characterized by its large-scale nuclear RNA-binding domain, is abundantly present in the “nuclear matrix” [23]. Additionally, SLTM possesses motifs for DNA binding, RNA binding and protein interaction, serving as both a transcriptional regulator and possessing epigenetic regulatory functions [50]. Previous studies have indicated that the generalized downregulation of mRNA synthesis by SLTM could be attributed to its inhibition of [^3^H]uridine incorporation into mRNA [24]. Nonetheless, the studies by Pedersen et al. [50], as well as our findings in this study, suggest that SLTM may have a stronger inhibitory impact on some genes, and the specific mechanisms behind this require additional investigation.

In summary, our study elucidated that increased DTL expression in HCC was closely related to adverse clinicopathological features and poor prognosis. DTL functions as an oncogene promoting the proliferation, metastasis, and sorafenib resistance of HCC cells. Mechanistically, HIF-1α activates DTL transcription by binding to HRE under hypoxia. DTL promotes the ubiquitination and degradation of SLTM, thereby relieving SLTM’s transcriptional repression of Notch1, leading to the activation of the Notch1 pathway and thus fulfilling its oncogenic function. The functional role of HIF-1α/DTL/SLTM axis in the proliferation, metastasis, and sorafenib resistance of HCC was confirmed by various models in vitro and in vivo. Therefore, we identified a promising therapeutic target for HCC intervention. Further research is necessary to determine the specific mechanisms between SLTM and the Notch1 pathway.

## Materials and methods

### Data collection and tissue specimens

In this study, three pairs of adjacent non-tumor, HCC, and PVTT tissue were collected with written informed consent and subjected to RNA-seq analysis. RNA-seq library preparation and sequencing were performed by GENEWIZ (Genewiz, Suzhou, China). Data on HCC gene expression and clinical features were retrospectively collected from TCGA (424 samples) dataset (http://cancergenome.nih.gov/) and GEO database (www.ncbi.nlm.nih.gov/geo), including GSE102079, GSE112790, GSE25097. Primary HCC tissue and corresponding non-tumor tissue were collected from 209 patients who underwent hepatectomy at the Sun Yat-sen University Cancer Center (Guangzhou, China) from May 2017 to May 2018. Surgical specimens were formalin-fixed and embedded in paraffin for IHC. All included patients were pathologically diagnosed as HCC with complete clinical information and follow-up data. The study protocol was authorized by the Ethics Committee of Sun Yat-sen University Cancer Center (G2022-068-01), and written informed consent was obtained from all participants. Clinical information of the HCC cohort is shown in Supplementary Table 1.

### Cells and chemicals

Huh-7, HCCLM-3, and HEK-293T cells were preserved by the Sun Yat-Sen University Cancer Center (Guangzhou, China) and cultured in DMEM (Gibco, Waltham, MA, USA) with 10% fetal bovine serum (FBS) (1050S, ABWBIO, Shanghai, China), and 1% penicillin–streptomycin (Gibco). Mycoplasma testing was performed every 3 months to ensure no mycoplasma contamination. All cells were incubated under 5% CO_2_ at 37 °C with 21% O_2_, while hypoxia experiments were performed under 1% O_2_ conditions. CoCl_2_ (60818, Sigma-Aldrich, Saint Louis, MO, USA) was diluted into a 150 mM stock solution with sterile water and added to the medium at the working concentration of 150 μM. KC7F2 (S7946, Selleck Chemicals, Shanghai, China) and Sorafenib (S7397, Selleck Chemicals) were dissolved in dimethyl sulfoxide (DMSO) to 10 mM stock solution. Tetracycline hydrochloride (T105493, Aladdin, Shanghai, China) was used at the working concentration of 1 μg/mL (in vitro) and 1 mg/mL (in vivo).

### RNA interference, plasmid constructions, and viral infections

siRNAs targeting DTL, HIF-1α, Notch1, and SLTM were provided by Ribo-Bio (Guangzhou, China). The overexpression vectors were constructed by cloning wide type (WT) HIF-1α cDNA sequences into pCDNA3.1 vectors. Likewise, the cDNA sequences of DTL, Notch1, and SLTM were cloned into pCDH-CMV-MCS-EF1-puro vectors with different primers. Lentiviral vectors expressing tetracycline-inducible shRNA against DTL were purchased from Youming-Bio (Guangzhou, China). The WT DTL and Notch1 promoter were amplified and cloned into PGL3-BASIC vector. The HRE deletion mutation vectors were generated by PCR based on the WT Vector. After the construction, all expression vectors were sequenced and verified by Ribo-Bio (Guangzhou, China). siRNA or plasmid was transfected into cells using lipofectamine 3000 (Invitrogen, Carlsbad, CA). The lentivirus containing overexpression or silencing plasmids was transfected into Huh-7 and HCCLM-3 cells. After 24 h, a puromycin-containing medium was used to screen for 2 weeks to obtain stable cell lines. Primer information and target sequences are listed in Supplementary Tables 4 and 5.

### Immunohistochemistry and scoring

The paraffin-embedded tissue sections were dewaxed with xylene and rehydrated with graded ethanol. The sections were soaked in sodium citrate buffer for antigen retrieval and incubated with 10% goat serum to block the non-specific binding sites. Subsequently, samples were incubated with primary antibodies against HIF-1α (1:200, Cell Signaling Technology, Boston), DTL (1:200; Abcam, Cambridge, UK), and *N*-cadherin (1:400, Proteintech, Wuhan, China) overnight at 4 °C, followed by incubation with corresponding HRP secondary antibodies for 1 h at room temperature. IHC scoring was independently assessed by two pathologists in a double-blinded manner.

### Quantitative real-time PCR (qRT-PCR)

Total RNA was extracted from cells using an RNA Purification Kit (EZBioscience, Shanghai, China). Reverse Transcription Kit (EZBioscience) was used to obtain cDNA; qRT-PCR was performed by 2×Color STBR Green qPCR Master Mix (EZBioscience) using CFX96/384 qPCR System (Bio-Rad, Hercules, CA). All primers used for qRT-PCR are listed in Supplementary Table 6.

### Immunofluorescence assay and western blots assay

Immunofluorescence staining was performed as described previously [51, 52]. Briefly, cells grown on coverslips were fixed with 4% paraformaldehyde for 15 min and permeabilized using 0.1% Triton X-100 for 10 min. After 1 h of blocking in 5% normal gout serum, coverslips were incubated with primary antibodies at 4 °C overnight. Fluorochrome-conjugated secondary antibodies were incubated for 1 h at room temperature; nuclei were stained with DAPI. Images were captured on a NIKON Eclipse Ti2-U.

For western blots, total proteins extracted from cells were separated by 10% sodium dodecyl sulfate-polyacrylamide gel electrophoresis (SDS-PAGE) and transferred to polyvinylidene fluoride (PVDF) membranes. The membranes were blocked by 5% non-fat milk for 1 h and then incubated overnight with primary antibodies at 4 °C. After washing, HRP-conjugated secondary antibodies were added and incubated for 40 min. Chemiluminescent signals were developed using Clarity Western ECL Substrate (Bio-Rad). Details of primary and secondary antibodies used for immunofluorescence and western blot are listed in Supplementary Table 7.

### Dual-luciferase reporter assay

Luciferase reporter plasmid of the DTL promotor and its mutant form were constructed as previously described. HEK-293T cells were seeded in 48-well plates and transfected with constructed firefly luciferase reporter plasmids (100 ng) and Renilla luciferase reporter plasmid (40 ng). Forty-eight hours after transfection, cells were lysed and luciferase activity was measured by a Dual-Luciferase Reporter Gene Assay Kit (Yeasen Biotechnology, Shanghai, China).

### Chromatin immunoprecipitation (ChIP)

To perform ChIP assays, an EZ-ChIP™ Kit (Merk Millipore, Billerica, MA) was used following the manufacturer’s instructions. Briefly, 3 × 10^7^ cells in each immunoprecipitation reaction were crosslinked with 1% formaldehyde, and 0.125 M glycine was added to terminate the reaction. Chromatin DNA was sonicated on ice to obtain 200–1000 bp fragments. The cell lysate was incubated overnight with HIF-1α or IgG antibodies and Protein A/G magnetic beads. After elution and decrosslinking, co-precipitated DNA was quantified by qRT-PCR.

### Immunoprecipitation and mass spectrometry

After lysing the cells with weak lysis buffer (Beyotime, Shanghai, China), the proteins were harvested and incubated with antibodies overnight at 4 °C on a rotating wheel. The mixtures were incubated with Sepharose-conjugated Protein A + G beads (Beyotime, Shanghai, China) for 4 h at 4 °C. After centrifugation and washing, the beads were boiled to release the proteins. The proteins were subjected to WB or LC–MS/MS analysis. The mass spectrometry analysis was performed by Huijun Biotechnology (Guangzhou, China).

### Cell-cycle analysis

All harvested cells were washed twice with ice-cold phosphate-buffered saline, fixed, and permeabilized with 70% ethanol at −20 °C overnight. Subsequently, samples were stained with propidium iodide (PI) (50 μg/mL) and RNase A (1 mg/mL) at room temperature for 30 min. Cell-cycle distribution was analyzed using PI staining by flow cytometry (Beckman CytoFlex).

### Cell Counting Kit-8 (CCK-8) assay

The HCC cells were seeded into a 96-well plate at the density of 1000/well. Five multiple wells were set up for each group. CCK-8 (Vazyme, Nanjing, China) solution (10 μL of reagent in 100 μL culture medium) was added into each well and incubated for 2 h. The absorbance value was measured at 450 nm.

### Clone formation assay

HCC cells (1 × 10^3^/well) were seeded in 6-well plates and cultured in a complete culture medium for 2 weeks. The colonies were fixed with methanol and stained with 0.1% crystal violet solution. Later, the number of colonies was counted using Image J software (version 6.0).

### Wound-healing assay

HCC cells were plated at a density of 1 × 10^6^/well in a 6-well plate. When the cell confluency reached 90%, a 200-μL pipette tip was used to scratch the cell monolayer. After washing with PBS, cells were incubated in a serum-free medium. The scratch closure was observed by an inverted microscope (Nikon Eclipse Ti2), and the scratch area was calculated with the Image J software (version 6.0).

### Transwell assays

Before seeding, cells were starved for 24 h, and the lower chamber was coated with fibronectin (20 μg/mL) for 2 h. The Matrigel (Corning, Shenzhen, China) was precoated for the invasion assay in the upper chamber. Then, cells were seeded into the upper chamber with 200 μL serum-free medium, and the bottom chambers were filled with 800 μL complete medium containing 10% FBS as a chemoattractant. After culturing for 24 h, the cells in the upper chamber were removed. Migrated cells were fixed with 4% paraformaldehyde and stained with 0.1% crystal violet solution. Cells on the bottom of the membrane were imaged and counted (five random fields per group).

### Tumor xenograft models

For the pulmonary metastasis model, 4-week-old male nude BALB/c mice were randomly assigned to each group. 2 × 10^6^ cells suspended in 200 μL PBS were injected into the lateral tail veins of mice. Mice were sacrificed 8 weeks later, and the lung tissue was collected, photographed, and fixed in 4% paraformaldehyde. For the orthotopic HCC implantation model, ~2 × 10^6^ HCC cells were suspended in a 40 μL DMEM–Matrigel mixture at a 1:1 ratio. Through a 1 cm midline incision in the upper abdomen under anesthesia, 6-week-old male nude BALA/c mice were orthotopically inoculated in the left hepatic lobe with a microsyringe. Six weeks later, all mice were sacrificed, and their livers and lungs were dissected, weighed, and fixed in 4% paraformaldehyde. HE staining was used to evaluate intrahepatic and lung metastasis. In addition, all mice in DTL knockdown and the control groups were fed with 1 mg/L tetracycline in the drinking water after inoculation of tumor cells to induce DTL knockdown in vivo. All animal studies were approved by the Sun Yat-sen University Cancer Center Animal Experimental Ethics Committee (L1020220210031).

### Statistical analysis

All statistical analysis and graphing were performed using the open-source R statistical software package (version 3.6.3) or GraphPad Prism (version 6.0). Data were shown as mean ± S.E.M. Chi-square test or Student’s *t*-test was used to compare the difference between the two groups. For multiple comparisons, one-way ANOVA with Tukey’s multiple comparisons test was used. Survival analysis was performed using Kaplan–Meier survival analysis. The Cox proportional hazards regression model was conducted for univariate and multivariate analysis. All analysis were two-tailed, with *P* < 0.05 considered significant: *P < 0.05; **P < 0.01; ***P < 0.001; ns nonsignificant.

## Supplementary information

Supplementary Table and Figure

Original Data

## Data Availability

The datasets used and/or analyzed during the current study are available from the corresponding author on reasonable request.
