# Supplementary material for: Hypoxia-induced DTL promotes the proliferation, metastasis, and sorafenib resistance of hepatocellular carcinoma through ubiquitin-mediated degradation of SLTM and subsequent Notch pathway activation
Source: Cell Death Dis. 2024 Oct 9;15(10):734. doi: 10.1038/s41419-024-07089-4 (PMC11464529; doi:10.1038/s41419-024-07089-4)
Supplement: Supplementary file 1 — Supplementary Table and Figure [file 41419_2024_7089_MOESM1_ESM.pdf]

Supplementary Table 1: Correlation analysis between the clinical features and DTL expression in HCC

| Characteristics of patients | | DTL Level | | P.overall |
|---|---|---|---|---|
| | | Low (N=169) | High (N=40) | |
| Sex | Male | 145 (85.8%) | 33 (82.5%) | 0.779 |
| | Female | 24 (14.2%) | 7 (17.5%) | |
| Age | <50 | 69 (40.8%) | 23 (57.5%) | 0.083 |
| | ≥50 | 100 (59.2%) | 17 (42.5%) | |
| Tumor size | <3.0cm | 70 (41.4%) | 14 (35.0%) | 0.572 |
| | ≥3.0cm | 99 (58.6%) | 26 (65.0%) | |
| Tumor differentiation | I/II | 96 (57.4%) | 19 (47.5%) | 0.339 |
| | III/IV | 93 (44.5%) | 21 (52.5%) | |
| Vascular invasion | Present | 58 (34.3%) | 26 (65.0%) | <**0.001***|
| | Absent | 111 (65.7%) | 14 (35.0%) | |
| Tumor number | <3 | 162 (95.9%) | 35 (87.5%) | 0.056 |
| | ≥3 | 7 (4.14%) | 5 (12.5%) | |
| AFP level | <400 | 124 (73.4%) | 23 (57.5%) | 0.074 |
| | ≥400 | 45 (26.6%) | 17 (42.5%) | |
| Liver cirrhosis | Present | 105 (62.1%) | 22 (55.0%) | 0.515 |
| | Absent | 64 (37.9%) | 18 (45.0%) | |
| Child-pugh class | A | 160 (94.7%) | 39 (97.5%) | 0.691 |
| | B | 9 (5.33%) | 1 (2.50%) | |
| TNM | I/II | 112 (66.3%) | 14 (35.0%) | <**0.001*** |
| | III/IV | 57 (33.7%) | 26 (65.0%) | |

HCC, hepatocellular carcinoma; AFP, alpha-fetoprotein; TNM tumor-node-metastasis. Significant values are in bold.

Supplementary Table 2: Multivariate Analyses of Predictors of Overall Survival

| Risk Factor | Univariate | | | Multivariate | | |
|---|---|---|---|---|---|---|
| | HR | 95%CI | *P* value | HR | 95%CI | *P* value |
| Sex | 2.466 | 0.764 - 7.956 | 0.131 | | | |
| Age | 0.5239 | 0.290-0.948 | **0.033*** | 0.649 | 0.352-1.197 | 0.166 |
| Liver cirrhosis | 1.063 | 0.586-1.931 | 0.840 | | | |
| Child-pugh class | 0.857 | 0.208-3.539 | 0.831 | | | |
| AFP level | 1.789 | 0.985-3.248 | 0.056 | | | |
| Tumor differentiation | 2.281 | 1.248-4.168 | **0.007*** | 1.823 | 0.978-3.398 | 0.059 |
| Tumor size | 3.434 | 1.598-7.376 | **0.002*** | 2.869 | 1.264-6.514 | **0.012*** |
| Tumor number | 1.677 | 0.600-4.684 | 0.324 | | | |
| Vascular invasion | 0.390 | 1.215-0.709 | **0.002*** | / | / | 1 |
| TNM | 2.623 | 1.444-4.765 | **0.002*** | / | / | 1 |
| DTL Level | 4.333 | 2.382-7.883 | **<0.001*** | 3.261 | 1.720-6.185 | **<0.001*** |

Abbreviations: HR, hazard ratio; AFP, Alpha-fetoprotein; TNM tumor-node-metastasis

*P value ≤ 0.05 was considered to indicate statistical significance.

Supplementary Table 3: Multivariate Analyses of Predictors of Disease-free Survival

| Risk Factor | Univariate | | | Multivariate | | |
|---|---|---|---|---|---|---|
| | HR | 95%CI | *P* value | HR | 95%CI | *P* value |
| Sex | 1.294 | 0.708-2.368 | 0.402 | | | |
| Age | 1.156 | 0.771-1.733 | 0.484 | | | |
| Liver cirrhosis | 0.137 | 0.768-1.712 | 0.505 | | | |
| Child-pugh class | 0.739 | 0.272-2.011 | 0.554 | | | |
| AFP level | 1.158 | 0.754-1.778 | 0.503 | | | |
| Tumor differentiation | 0.267 | 0.880-1.938 | **0.184*** | 1.869 | 1.003-3.485 | **0.049*** |
| Tumor size | 1.647 | 1.080-2.510 | **0.021*** | 2.806 | 1.232-6.391 | **0.014*** |
| Tumor number | 1.355 | 0.628-2.928 | 0.439 | | | |
| Vascular invasion | 0.363 | 0.243-0.542 | **<0.001*** | / | / | 1 |
| TNM | 2.675 | 1.795-3.986 | **<0.001*** | / | / | 1 |
| DTL Level | 2.205 | 1.397-3.480 | **<0.001*** | 3.60 | 1.942-6.659 | **<0.001*** |

Abbreviations: HR, hazard ratio; AFP, Alpha-fetoprotein; TNM tumor-node-metastasis

*P value ≤ 0.05 was considered to indicate statistical significance.

Supplementary Table 4: Primer sequences for plasmid construction

| Primer name | Sequence |
|---|---|
| DTL WT Forward | 5’- GTACTCGAGATGCTCTTCAATTCGGTGCT -3’ |
| DTL WT Reverse | 5’- GTAGGATCCCTATAATTCTGTTGAGTGTT -3’ |
| HIF-1α WT Forward | 5’-GTAGGTACCAATAAGTGGTGGTTACTCAGCAC-3’ |
| HIF-1α WT Reverse | 5’-GTAGGGCCCCCTGGTCCACAGAAGATGTTTA-3’ |
| Notch1 WT Forward | 5’- CTCTAGAGATGACTAGTGCCTCGGCC-3’ |
| Notch1 WT Reverse | 5’-GGAATTCCTGGAAGCCAGATCACCATCAG-3’ |
| shDTL NC Forward | 5’- CCGGACGCATGCATGCTCAGTGCTTCTCG AGAAGCACTGAGCATGCATGCGTTTTTG -3’ |
| shDTL NC Reverse | 5’- AATTCAAAAACGCATGCATGCTCAGTGC TTCTCGAGAAGCACTGAGCATGCATGCGT -3’ |
| shDTL1 Forward | 5’- CCGGCCTGGTGAACTTAAACTTGTTCTCG AGAACAAGTTTAAGTTCACCAGGTTTTTG -3’ |
| shDTL1 Reverse | 5’- AATTCAAAAACCTGGTGAACTTAAACTTG TTCTCGAGAACAAGTTTAAGTTCACCAGG -3’ |
| shDTL2 Forward | 5’- CCGGGCCTAGTAACAGTAACGAGTACTCG AGTACTCGTTACTGTTACTAGGCTTTTTG -3’ |
| shDTL2 Reverse | 5’- AATTCAAAAAGCCTAGTAACAGTAACGAG TACTCGAGTACTCGTTACTGTTACTAGGC -3’ |
| DTL promoter Forward | 5’-CTAGCTAGCTAGGTATCAGAATGAGGAGGAAAGTG-3’ |
| DTL promoter Reverse | 5’- CCGCTCGAGCGGGGGAGAACTCAGAAGCTGAG-3’ |
| Notch1 promoter Forward | 5’-GGCTAGCCATGCAAACCAGGCTCTGCGG-3’ |
| Notch1 promoter Reverse | 5’-GGAATTCCACCGGCTGCCCTCTG-3’ |
| DTL-CHIP-P1 Forward | 5’- GAGCTCGCTACTTGTTTAGC-3’ |
| DTL-CHIP-P1 Reverse | 5’- CAGGAACTGGATGCCAACTC-3’ |
| DTL-CHIP-P2 Forward | 5’- GTCAAGCCCTGTTACGCATG-3’ |
| DTL-CHIP-P2 Reverse | 5’- CCAAGTTCAGCAGAAATCGC-3’ |
| DTL-P1-mutant Forward | 5’- GCTCCCCGCCCAGCGAATGCGCCTATCCGTC-3’ |
| DTL-P1-mutant Reverse | 5’- GACGGATAGGCGCATTCGCTGGGCGGGGAGC-3’ |

| | |
|---|---|
| DTL-P2-mutant Forward | 5’- GACTCTGAGGCGCCTCGGAAGCCAATC-3’ |
| DTL-P2-mutant Reverse | 5’- GATTGGCTTCCGAGGCGCCTCAGAGTC-3’ |
| 3xFlag-DTL Forward | 5’-GTACTCGAGATGGACTACAAAGACCATGACGGTG ATTATAAAGATCATGACATCGACTACAAGGATGACG ATGACAAGATGCTCTTCAATTCGGTGCT-3’ |
| 2xHA-SLTM Forward | 5’-CTAGCTAGCTAGATGTACCCATACGATGTTCCAGATT ACGCTTACCCATACGATGTTCCAGATTACGCTATG ATTGTGCGCAGCGCTGC-3’ |
| 2xHA-SLTM Reverse | 5’- GTCGACTTCCAGGTGTGGATTTTTATTTTCAC -3’ |
| 1xFlag-DTL 1-398aa Forward | 5’- GTAGCTAGCATGGATTACAAGGACGACGATGACAAG ATGCTCTTCAATTCGGTGCT-3’ |
| 1xFlag-DTL 1-398aa Reverse | 5’- GTAGGATCCCTAGGTTTCTCCTCTAAGCCTCT-3’ |
| 1xFlag-DTL 1-448aa Reverse | 5’-GTAGGATCCCTATGCGGATGACGGGGAAG-3’ |
| 1xFlag-DTL 1-498aa Reverse | 5’- GTAGGATCCCTATTGAAAGATGAAGGTGGCTT-3’ |
| 1xFlag-DTL 1-598aa Reverse | 5’- GTAGGATCCCTAAGGTCTTCCTGGTTACCAGC-3’ |
| 1xFlag-DTL 398-730aa Forward | 5’- GTAGCTAGCATGGATTACAAGGACGACGATGACAAG GGAGGTGATAAACTTTCCAC-3’ |
| 1xFlag-DTL 398-730aa Reverse | 5’-GTAGGATCCCTATAATTCTGTTGAGTGTT-3’ |

Supplementary Table 5: Target sequence of siRNA

| Primer name | Sequence |
|---|---|
| DTL Si-1 Forward | 5’- GGUUCCUGGUGAACUUAAACU-3’ |
| DTL Si-1 Reverse | 5’- UUUAAGUUCACCAGGAACCCA-3’ |
| DTL Si-2 Forward | 5’- GGCUCUACUUUAUUUGCUAAU-3’ |
| DTL Si-2 Reverse | 5’- UAGCAAAUAAAGUAGAGCCAG-3’ |
| HIF-1α Si-1 Forward | 5’- GGAAAUGAGAGAAAUGCUUDTDT-3’ |
| HIF-1α Si-1 Reverse | 5’- AAGCAUUUCUCUCAUUUCCDTDC-3’ |
| HIF-1α Si-2 Forward | 5’- GCUGGAGACACAAUCAUAUDTDT-3’ |
| HIF-1α Si-2 Reverse | 5’- AUAUGAUUGUGUCUCCAGCDGDG-3’ |
| Notch1 Si-1 Forward | 5’-GGAGCAUGUGUAACAUCAADTDT-3’ |
| Notch1 Si-1 Reverse | 5’-UUGAUGUUACACAUGCUCCDTDT-3’ |
| Notch1 Si-2 Forward | 5’-GUCCAGGAAACAACUGCAADTDT-3’ |
| Notch1 Si-2 Reverse | 5’-UUGCAGUUGUUUCCUGGACDTDT-3’ |
| SLTM Si-1 Forward | 5’- GCUGUGUGGUAGUGGUUUAAU-3’ |
| SLTM Si-1 Reverse | 5’- UAAACCACUACCACACAGCUG-3’ |
| SLTM Si-2 Forward | 5’- GGAAGUACAAGUAGUACUAGU-3’ |
| SLTM Si-2 Reverse | 5’- UAGUACUACUUGUACUUCCUU-3’ |

Supplementary Table 6: Primers for Quantitative Real-time PCR

| Primer name | Sequence |
|---|---|
| DTL Forward | 5’- CCCTTCAAAACCCAAGAAGA -3’ |
| DTL Reverse | 5’- GGTTCTTGTCGATAAGCAGT -3’ |
| HIF-1α Forward | 5’-CAGCGAAGCTTTTTTCTCAGAATGA-3’ |
| HIF-1α Reverse | 5’- CACAAATCAGCACCAAGC-3’ |
| GAPDH Forward | 5’- TGTGTCCGTCGTGGATCTGA -3’ |
| GAPDH Reverse | 5’- TTGCTGTTGAAGTCGCAGGAG -3’ |
| NOTCH Forward | 5’-GAGGCGTGGCAGACTATGC-3’ |
| NOTCH Reverse | 5’-CTTGTACTCCGTCAGCGTGA-3’ |
| Fibronectin Forward | 5’-GGAGGAAGCCGAGGTTTTAAC-3’ |
| Fibronectin Reverse | 5’-ACGCTCATAAGTGTCACCCA-3’ |
| N-cadherin Forward | 5’-TCGATTGGTTTGACCACGG-3’ |
| N-cadherin Reverse | 5’-GACGGTTCGCCATCCAGAC-3’ |
| E-cadherin Forward | 5’-AGTGGGCACAGATGGTGTGA-3’ |
| E-cadherin Reverse | 5’-TAGGTGGAGTCCCAGGCGTA-3’ |
| Vimentin Forward | 5’-TGTCCAAATCGATGTGGATGTTTC-3’ |
| Vimentin Reverse | 5’-TTGTACCATTCTTCTGCCTCCTG-3’ |
| Slug Forward | 5’-GACCCTGGTTGCTTCAAGGA-3’ |
| Slug Reverse | 5’-TGTTGCAGTGAGGGCAAGAA-3’ |
| SNAIL Forward | 5’-TCGGAAGCCTAACTACAGCGA-3’ |
| SNAIL Reverse | 5’-AGATGAGCATTGGCAGCGAG-3’ |
| SLTM Forward | 5’-AGACAGGCGAGAAGTACGAG-3’ |
| SLTM Reverse | 5’-CTGGTGGGTCTGGAAGGATT-3’ |
| β-actin Forward | 5’-CATGTACGTTGCTATCCAGGC-3’ |
| β -actin Reverse | 5’-CTCCTTAATGTCACGCACGAT-3’ |

Supplementary Table 7: List of the primary and secondary antibodies used in this study

| Antigen | Species | Source | Catalog |
|---|---|---|---|
| DTL | Rabbit | Abcam | ab174385 |
| HIF-1α | Rabbit | CST | #3716 |
| GAPDH | Rabbit | Proteintech | 10494 |
| β-tubulin | Rabbit | CST | #2128 |
| Fibronectin | Mouse | Proteintech | 15613 |
| N-cadherin | Rabbit | Affinity | AF5239 |
| E-cadherin | Rabbit | Affinity | AF0131 |
| Vimentin | Rabbit | Proteintech | 10366 |
| Slug | Rabbit | Proteintech | 12129 |
| Snail | Rabbit | Proteintech | 13099 |
| Notch1 | Rabbit | Abcam | EP1238Y |
| FLAG | Mouse | Proteintech | 66008 |
| HA | Rabbit | Proteintech | 81290 |
| SLTM | Rabbit | Novus | NBP1-47300 |
| Ubiquitin | Rabbit | Proteintech | 10201 |
| HRP-conjugated Goat Anti-Rabbit IgG (H+L) | Goat | Proteintech | SA00001-2 |
| HRP-conjugated Affinipure Goat Anti-Mouse IgG (H+L) | Goat | Proteintech | SA00001-1 |
| Goat Anti-Rabbit IgG (H+L) Flour 488-conjugated | Goat | Affinity | S0018 |
| Goat Anti-Rabbit IgG (H+L) Flour 594-conjugated | Goat | Affinity | S0006 |

Supplementary Figure 1: (A) Heatmap of DEGs for GSE102079, GSE112790, and GSE25097 datasets from GEO, respectively. (B-D). Volcano plot of hepatocellular carcinoma (HCC) vs. adjacent nontumor, portal vein tumor thrombosis (PVTT) vs. adjacent nontumor, and PVTT vs HCC, respectively.

Supplementary Figure 2: (A) DTL mRNA levels between HCC samples and matched liver tissue (n=50) from the TCGA database. (B) Kaplan-Meier survival analysis of OS for HCC patients with high and low expression of DTL from TCGA database. (C-E) DTL mRNA levels among different histological grade (C), pathologic T stage (D), and pathologic N stage (E) in the TCGA-LIHC cohort. (F) The capacity to identify tumor or normal tissue sources of DTL in the TCGA-LIHC cohort. *$P < 0.05$, **$P < 0.01$, ***$P < 0.001$, ns, no significance

Supplementary Figure 3: Spearman correlation analysis between DTL and pathway score. [52] The signal pathway positively correlated with the overexpression of DTL.

Supplementary Figure 4: (A) The mRNA level of HIF-1α and DTL was determined after HIF-1α-siRNA transfection or KC7F2 (40 μM) treatment under hypoxia (1% O2) in HCCLM-3 cells. (B) The protein level of HIF-1α and DTL was quantified after HIF-1α-siRNA transfection or KC7F2 (40 μM) treatment under hypoxia (1% O2) in Huh-7 and HCCLM-3 cells. (C) The effectiveness of doxycycline-induced DTL knockdown in Huh-7 and HCCLM-3 cells was detected in various concentrations. Knockdown of

DTL was mediated by treatment with 1.0 μg/mL doxycycline for 48 h in further experiments. (D) Representative Flow Cytometry Analysis of Cell Cycle Distribution in Huh-7 and HCCLM-3 Cells Following Overexpression and Knockdown of DTL.

Supplementary Figure 5: (A) Transwell assays were performed to evaluate the invasion ability of DTL overexpression and knockdown HCC cells. (B) Representative images of wound healing assay in HCC cells. (C) Representative images of lung tissues in tail vein tumor metastasis mouse model from DTL knockdown and negative control (NC) groups. (D) Representative images of liver tissues in orthotopic liver transplantation model from DTL knockdown and negative control (NC) groups. (E) Liver weight in orthotopic liver transplantation model. (F) Representative images of intrahepatic metastasis nodules in HE-stained sections. Left panel: the number of intrahepatic metastasis nodules in the DTL overexpression group. (G and H) Representative images of lung tissues in orthotopic liver transplantation model from DTL overexpression (G) and knockdown (H) groups.

Supplementary Figure 6: (A) The immunofluorescence results of Notch1 and N-cadherin expression in DTL overexpressing or knockdown Huh-7 cells. (B) The knockdown efficiency of Notch1 by siRNA was detected by qRT-PCR in HCCLM-3 cells. (C) The functional role of Notch1 in the DTL-induced proliferation of HCCLM-3 cells was detected by Cell Counting Kit-8 (CCK8) (D). The functional role of Notch1 in DTL-induced metastasis of HCCLM-3 cells was detected by wound-healing assays.

(E) Representative images of transwell assays demonstrating the effect of Notch1 knockdown on the metastasis and invasiveness of DTL-overexpressing Huh-7 Cells. (F) Transwell assays representative images and statistical outcomes assessing the impact of Notch1 knockdown on the metastasis and invasiveness properties of HCCLM-3 cells with DTL overexpression. (G) CCK-8 assay evaluating HCCLM-3 cells viability under DTL overexpression or hypoxia with Notch1 knockdown and sorafenib (3μM) treatment. *P < 0.05, **P < 0.01, ***P < 0.001, ns, no significance

Supplementary Figure 7: (A) Validation of 3xFlag-DTL overexpression in Huh-7 cells using Western blot. (B) The top 20 proteins interacting with DTL, identified through liquid chromatography-tandem mass spectrometry (LC-MS/MS) analysis.

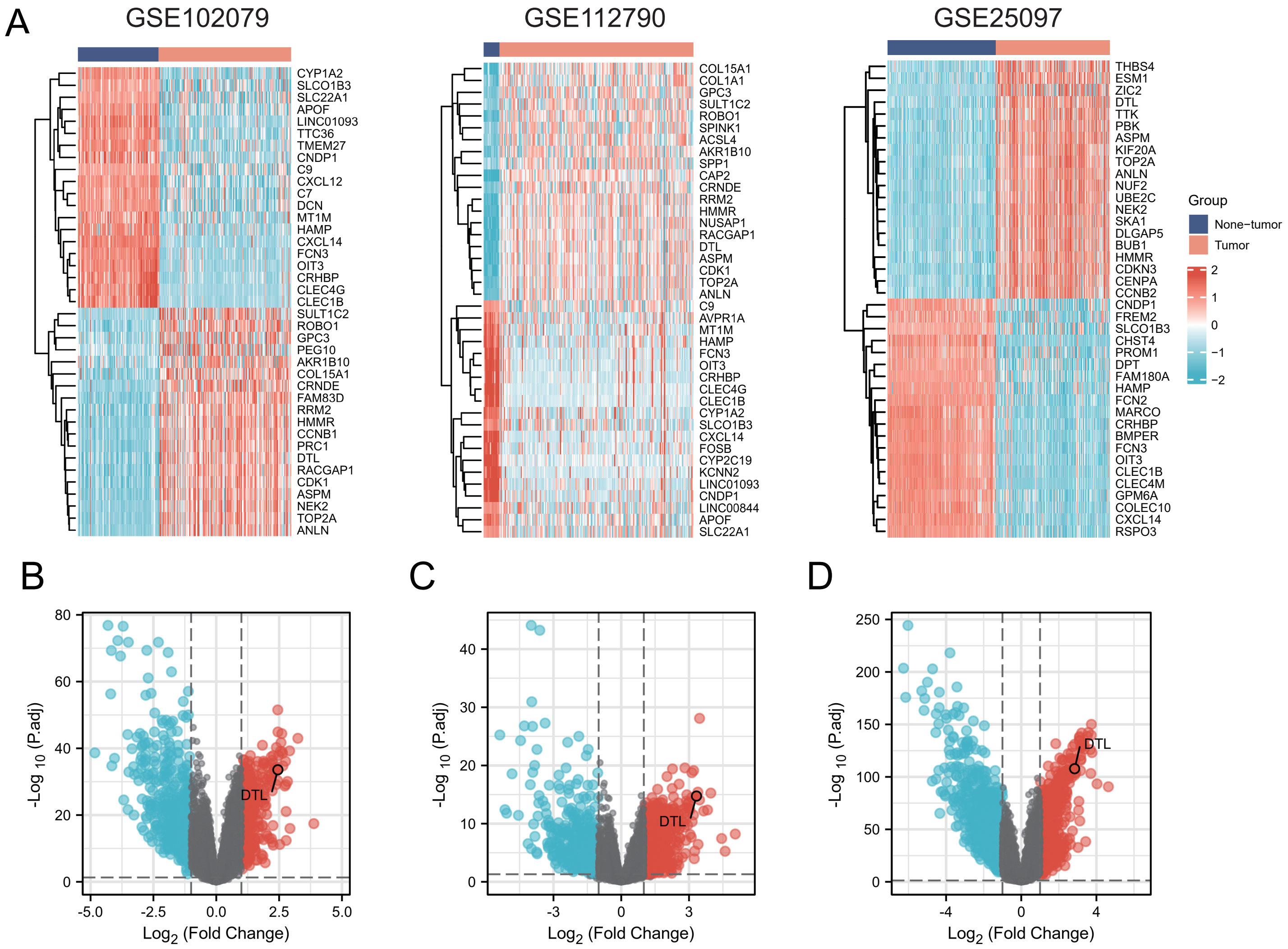
A
GSE102079
GSE112790
GSE25097
CYP1A2
SLCO1B3
SLC22A1
APOF
LINC01093
TTC36
TMEM27
CNDP1
C9
CXCL12
C7
DCN
MT1M
HAMP
CXCL14
FCN3
OIT3
CRHBP
CLEC4G
CLEC1B
SULT1C2
ROBO1
GPC3
PEG10
AKR1B10
COL15A1
CRNDE
FAM83D
RRM2
HMMR
CCNB1
PRC1
DTL
RACGAP1
CDK1
ASPM
NEK2
TOP2A
ANLN
COL15A1
COL1A1
GPC3
SULT1C2
ROBO1
SPINK1
ACSL4
AKR1B10
SPP1
CAP2
CRNDE
RRM2
HMMR
NUSAP1
RACGAP1
DTL
ASPM
CDK1
TOP2A
ANLN
C9
AVPR1A
MT1M
HAMP
FCN3
OIT3
CRHBP
CLEC4G
CLEC1B
CYP1A2
SLCO1B3
CXCL14
FOSB
CYP2C19
KCNN2
LINC01093
CNDP1
LINC00844
APOF
SLC22A1
THBS4
ESM1
ZIC2
DTL
TTK
PBK
ASPM
KIF20A
TOP2A
ANLN
NUF2
UBE2C
NEK2
SKA1
DLGAP5
BUB1
HMMR
CDKN3
CENPA
CCNB2
CNDP1
FREM2
SLCO1B3
CHST4
PROM1
DPT
FAM180A
HAMP
FCN2
MARCO
CRHBP
BMPER
FCN3
OIT3
CLEC1B
CLEC4M
GPM6A
COLEC10
CXCL14
RSPO3
Group
None−tumor
Tumor
2
1
0
−1
−2
B
C
D
-Log 10 (P.adj)
Log2 (Fold Change)
DTL
80
60
40
20
0
-5.0
-2.5
0.0
2.5
5.0
40
30
20
10
0
-3
0
3
250
200
150
100
50
0
-4
0
4

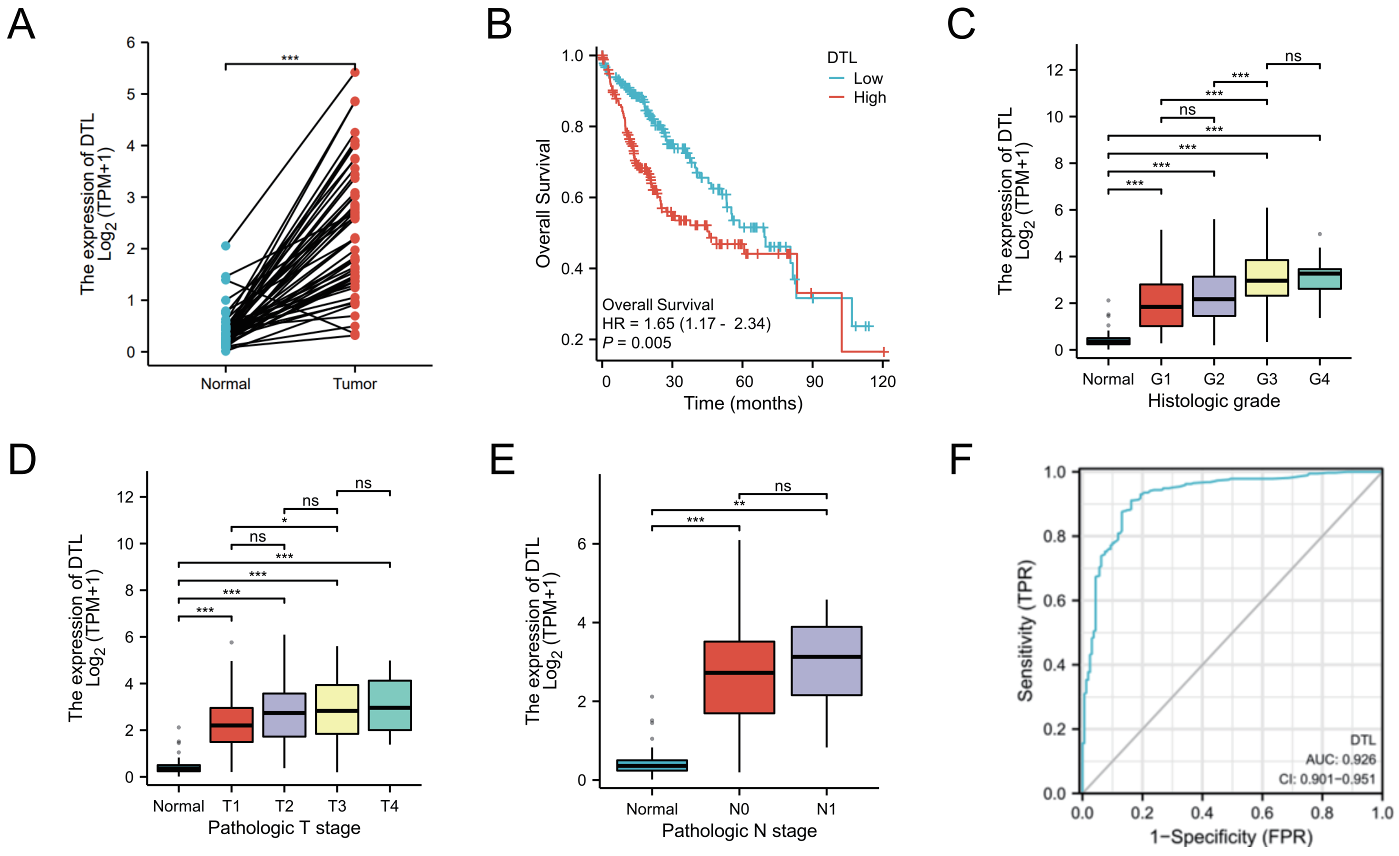
A
The expression of DTL
Log2 (TPM+1)
***
Normal
Tumor
B
DTL
Low
High
Overall Survival
Overall Survival
HR = 1.65 (1.17 - 2.34)
P = 0.005
Time (months)
C
The expression of DTL
Log2 (TPM+1)
ns
***
Normal
G1
G2
G3
G4
Histologic grade
D
The expression of DTL
Log2 (TPM+1)
ns
*
***
Normal
T1
T2
T3
T4
Pathologic T stage
E
The expression of DTL
Log2 (TPM+1)
ns
**
***
Normal
N0
N1
Pathologic N stage
F
Sensitivity (TPR)
1−Specificity (FPR)
DTL
AUC: 0.926
CI: 0.901−0.951

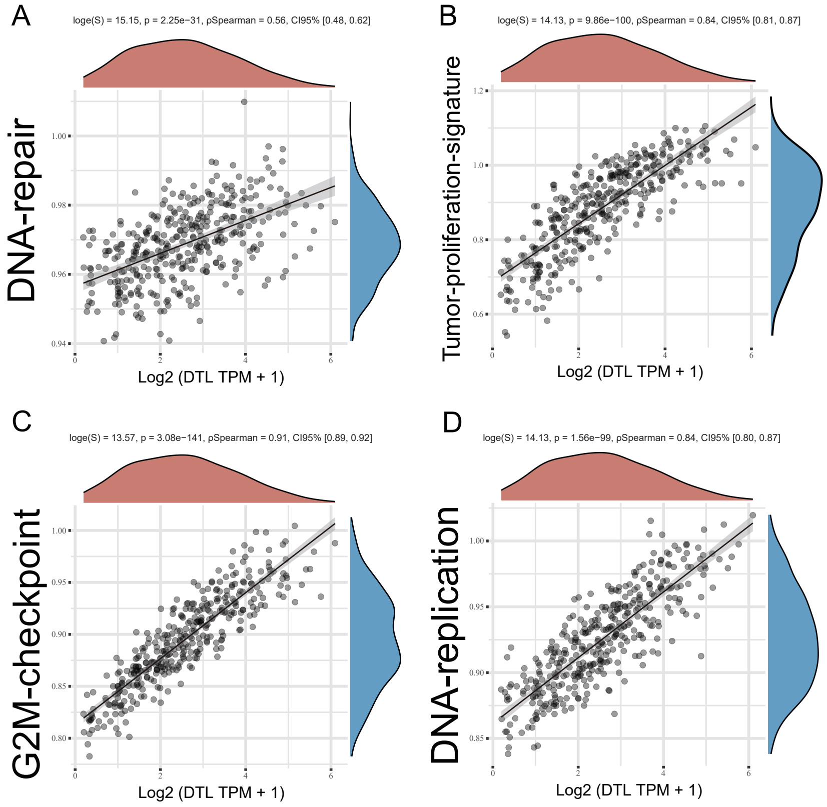
A
loge(S) = 15.15, p = 2.25e−31, ρSpearman = 0.56, CI95% [0.48, 0.62]
DNA-repair
Log2 (DTL TPM + 1)
B
loge(S) = 14.13, p = 9.86e−100, ρSpearman = 0.84, CI95% [0.81, 0.87]
Tumor-proliferation-signature
Log2 (DTL TPM + 1)
C
loge(S) = 13.57, p = 3.08e−141, ρSpearman = 0.91, CI95% [0.89, 0.92]
G2M-checkpoint
Log2 (DTL TPM + 1)
D
loge(S) = 14.13, p = 1.56e−99, ρSpearman = 0.84, CI95% [0.80, 0.87]
DNA-replication
Log2 (DTL TPM + 1)

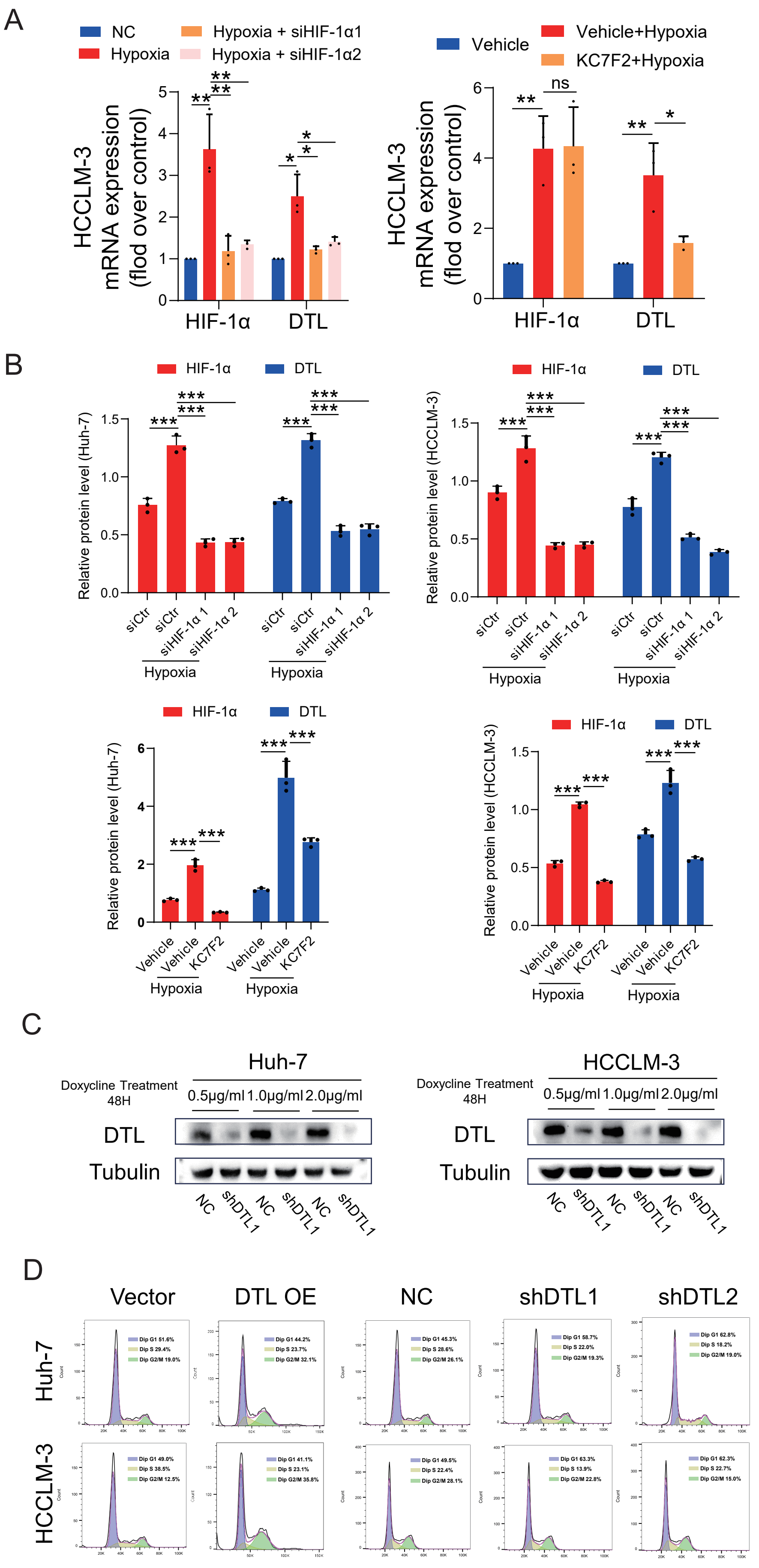
A
NC
Hypoxia
Hypoxia + siHIF-1α1
Hypoxia + siHIF-1α2
HCCLM-3 mRNA expression (flod over control)
HIF-1α
DTL
Vehicle
Vehicle+Hypoxia
KC7F2+Hypoxia
ns
B
HIF-1α
DTL
Relative protein level (Huh-7)
Relative protein level (HCCLM-3)
siCtr
siHIF-1α 1
siHIF-1α 2
Hypoxia
Vehicle
KC7F2
C
Huh-7
HCCLM-3
Doxycline Treatment 48H
0.5μg/ml
1.0μg/ml
2.0μg/ml
DTL
Tubulin
NC
shDTL1
D
Vector
DTL OE
NC
shDTL1
shDTL2
Huh-7
HCCLM-3

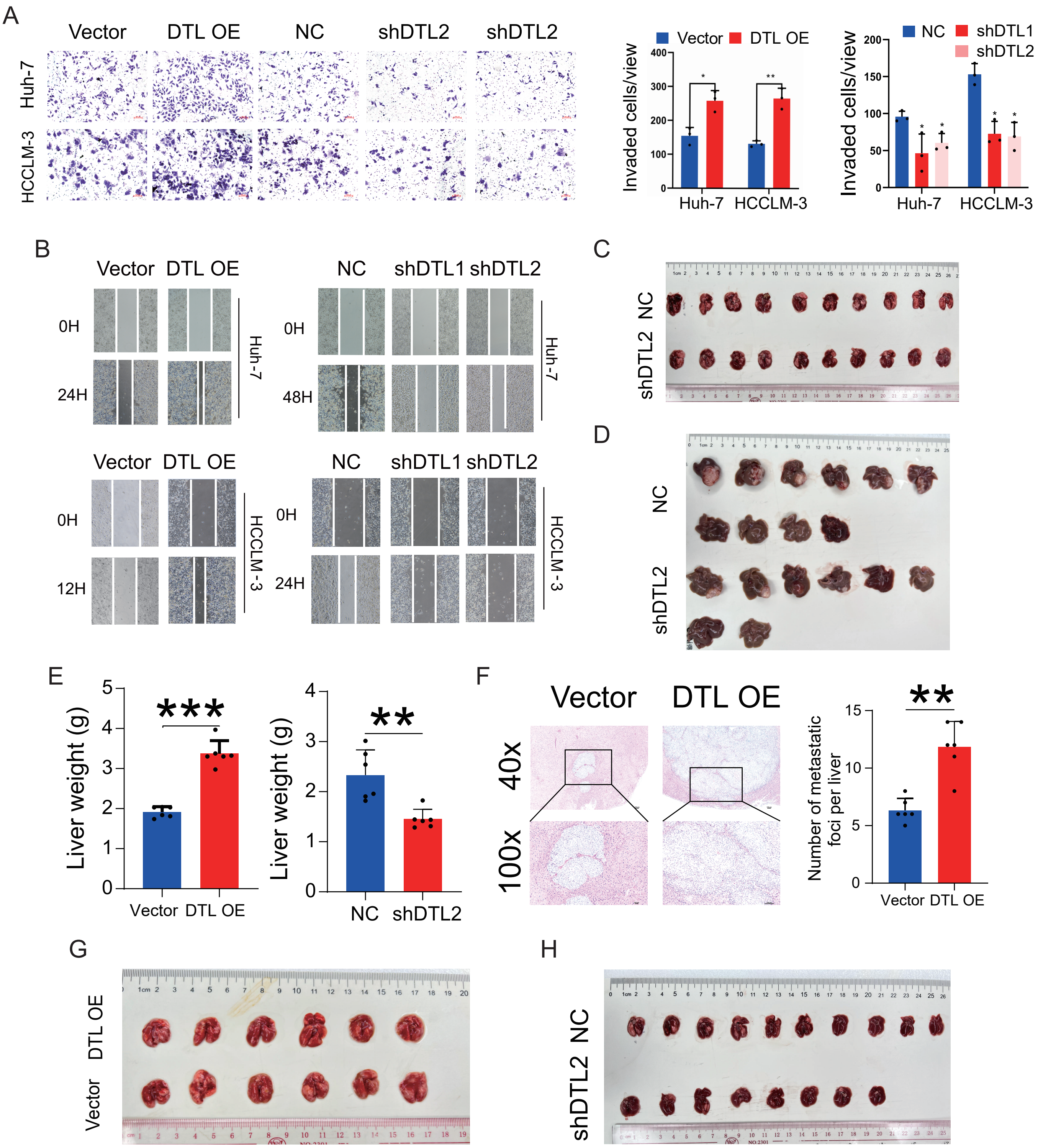
A
Vector
DTL OE
NC
shDTL2
shDTL2
Huh-7
HCCLM-3
Vector
DTL OE
Invaded cells/view
Huh-7
HCCLM-3
NC
shDTL1
shDTL2
Invaded cells/view
Huh-7
HCCLM-3
B
Vector
DTL OE
0H
24H
Huh-7
NC
shDTL1
shDTL2
0H
48H
Huh-7
Vector
DTL OE
0H
12H
HCCLM-3
NC
shDTL1
shDTL2
0H
24H
HCCLM-3
C
shDTL2 NC
D
NC
shDTL2
E
Liver weight (g)
Vector
DTL OE
Liver weight (g)
NC
shDTL2
F
Vector
DTL OE
40x
100x
Number of metastatic foci per liver
Vector
DTL OE
G
DTL OE
Vector
H
shDTL2 NC

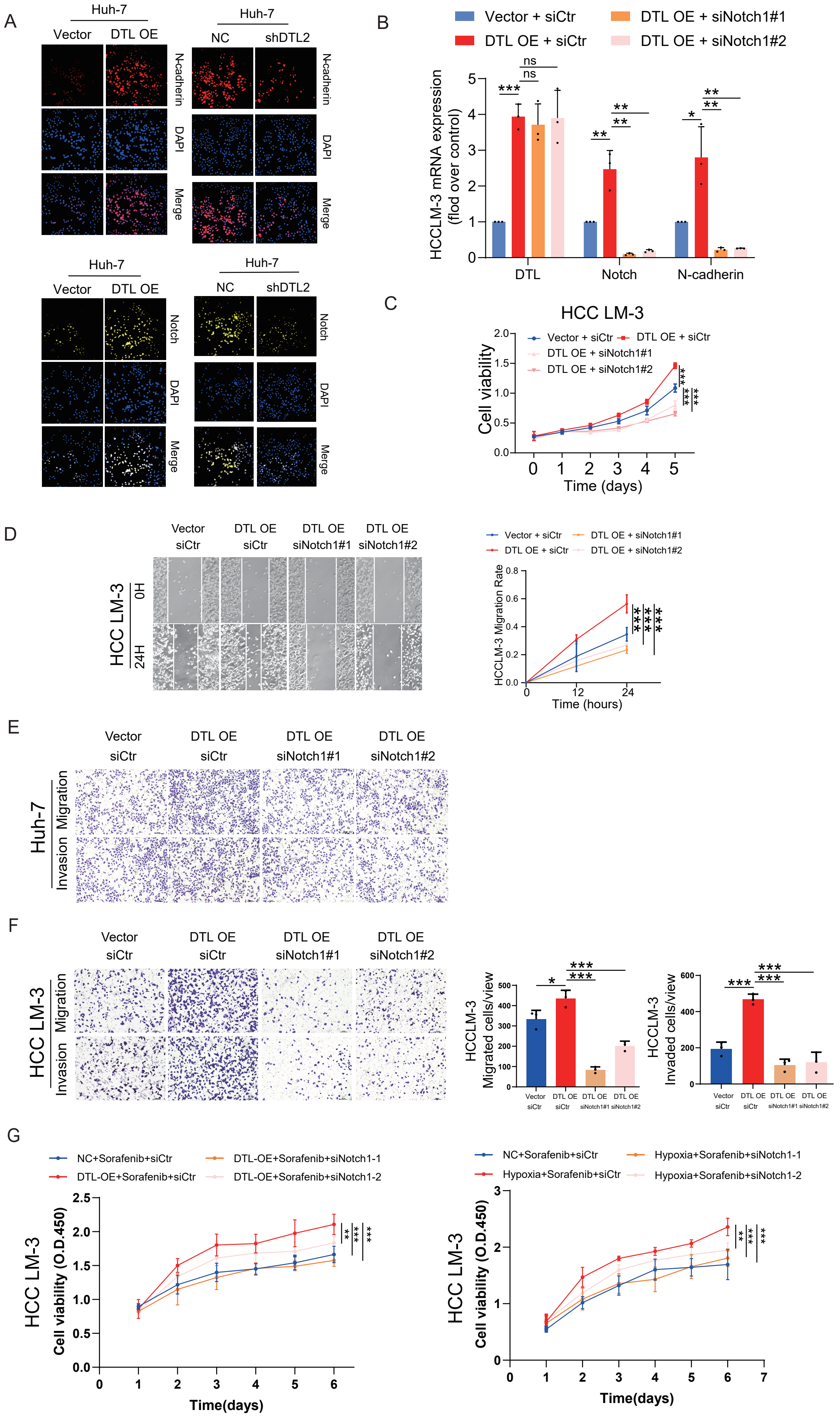

A
Huh-7
Vector
DTL OE
NC
shDTL2
N-cadherin
DAPI
Merge
Notch
B
Vector + siCtr
DTL OE + siCtr
DTL OE + siNotch1#1
DTL OE + siNotch1#2
HCCLM-3 mRNA expression (flod over control)
DTL
Notch
N-cadherin
C
HCC LM-3
Cell viability
Time (days)
D
HCC LM-3
0H
24H
HCCLM-3 Migration Rate
Time (hours)
E
Migration
Invasion
F
HCCLM-3 Migrated cells/view
HCCLM-3 Invaded cells/view
G
NC+Sorafenib+siCtr
DTL-OE+Sorafenib+siCtr
DTL-OE+Sorafenib+siNotch1-1
DTL-OE+Sorafenib+siNotch1-2
Hypoxia+Sorafenib+siCtr
Hypoxia+Sorafenib+siNotch1-1
Hypoxia+Sorafenib+siNotch1-2
Cell viability (O.D.450)
Time(days)

A

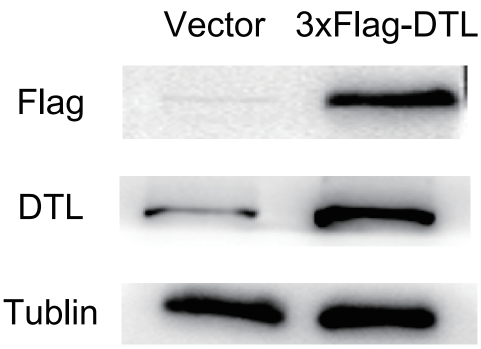


B Mass spectrometric identification of the proteins bound to DTL.

| Number | Citable Accession | Gene name |
|---|---|---|
| 1 | Q14684 | RRP1B |
| 2 | Q8TDD1 | DDX54 |
| 3 | P46013 | MKI67 |
| 4 | Q9NZJ0 | DTL |
| 5 | O00560 | SDCBP |
| 6 | O15213 | WDR46 |
| 7 | P55081 | MFAP1 |
| 8 | Q1ED39 | KNOP1 |
| 9 | Q06787 | FMR1 |
| 10 | Q9NWH9 | SLTM |
| 11 | Q13595 | TRA2A |
| 12 | Q7Z6E9 | RBBP6 |
| 13 | Q9NWB6 | ARGLU1 |
| 14 | Q8NC51 | SERBP1 |
| 15 | P46778 | RPL21 |
| 16 | Q9BYG3 | NIFK |
| 17 | Q13601 | KRR1 |
| 18 | P62910 | RPL32 |
| 19 | P62136 | PPP1CA |
| 20 | Q8NI27 | THOC2 |
