# Supplementary material for: Hypoxia-induced DTL promotes the proliferation, metastasis, and sorafenib resistance of hepatocellular carcinoma through ubiquitin-mediated degradation of SLTM and subsequent Notch pathway activation
Source: Cell Death Dis. 2024 Oct 9;15(10):734. doi: 10.1038/s41419-024-07089-4 (PMC11464529; doi:10.1038/s41419-024-07089-4)

Full unedited blots for figure 2E

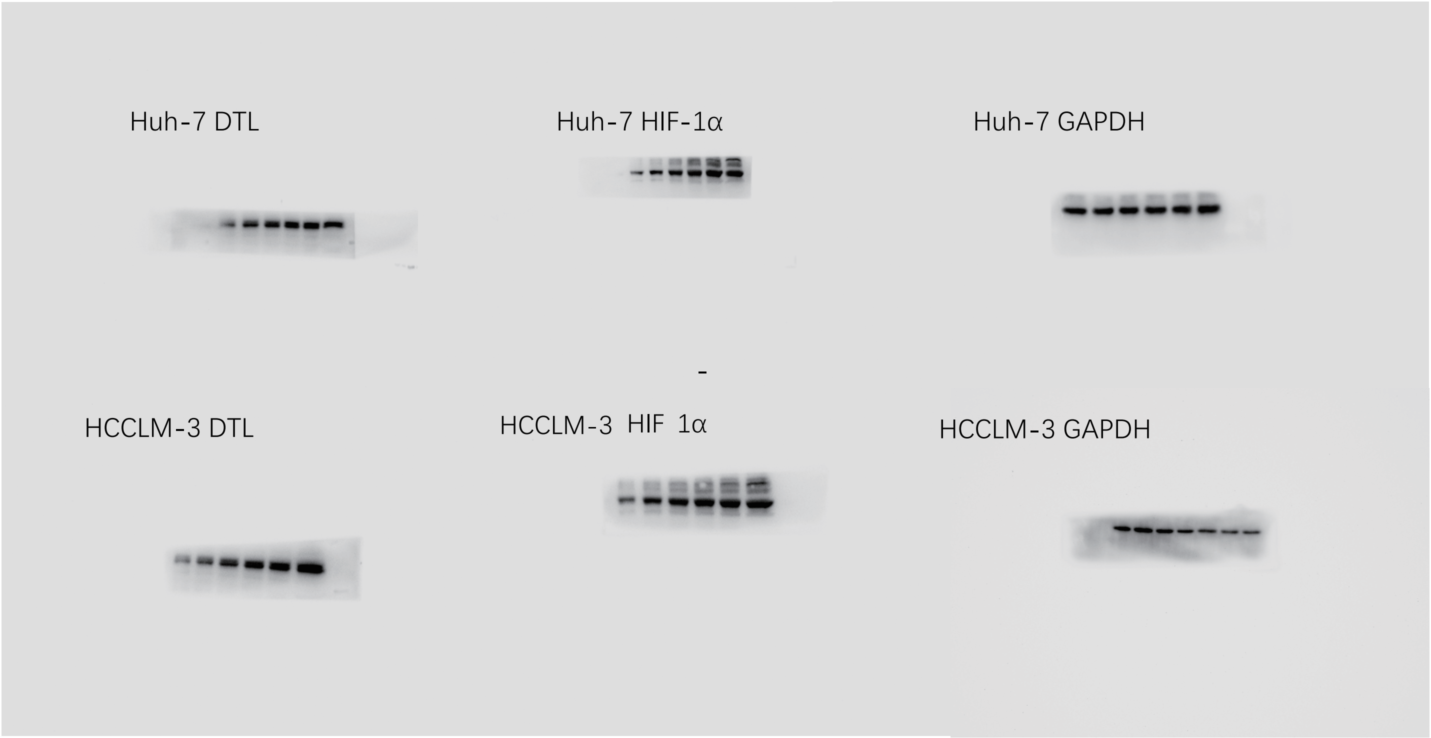

Full unedited blots for figure 3B

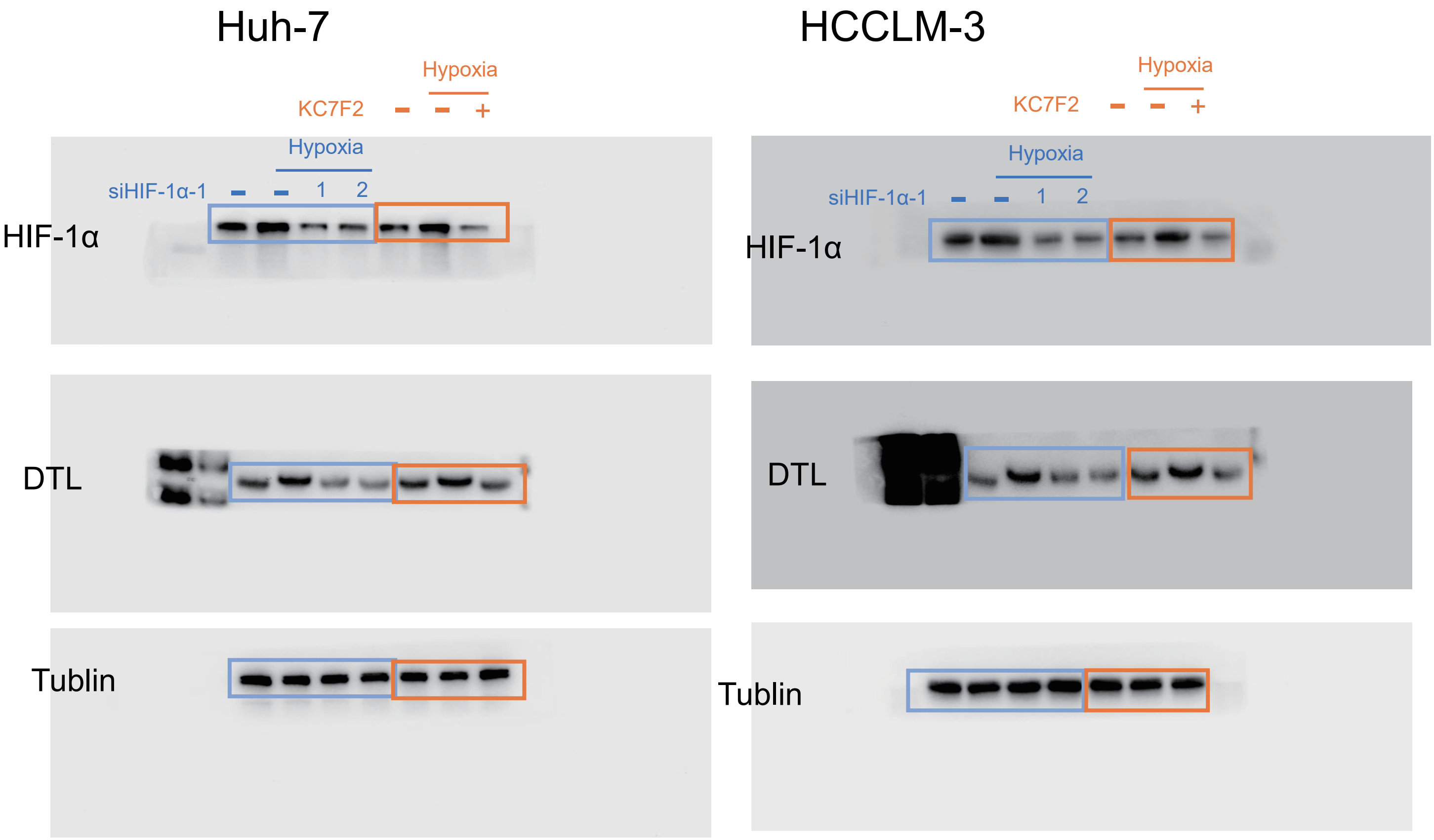

Full unedited blots for figure 4B

Huh-7 DTL

Vector DTL OE

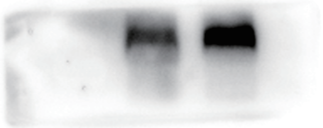

NC shDTL1 shDTL2

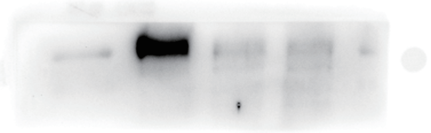

HCCLM-3 DTL

Vector DTL OE

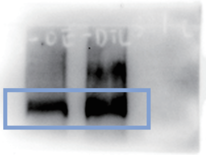

NC shDTL1 shDTL2

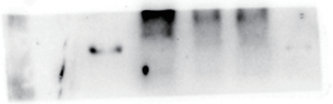

Huh-7 Tublin

Vector DTL OE

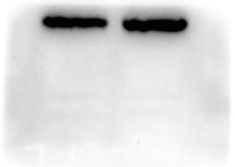

NC shDTL1 shDTL2

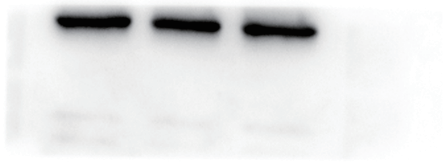

HCCLM-3 Tublin

Vector

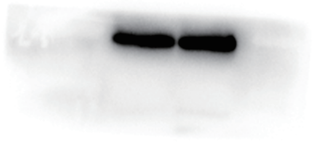

NC shDTL1 shDTL2

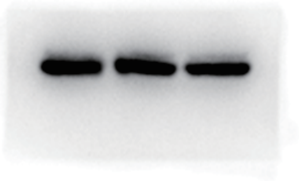

Full unedited blots for figure 7D

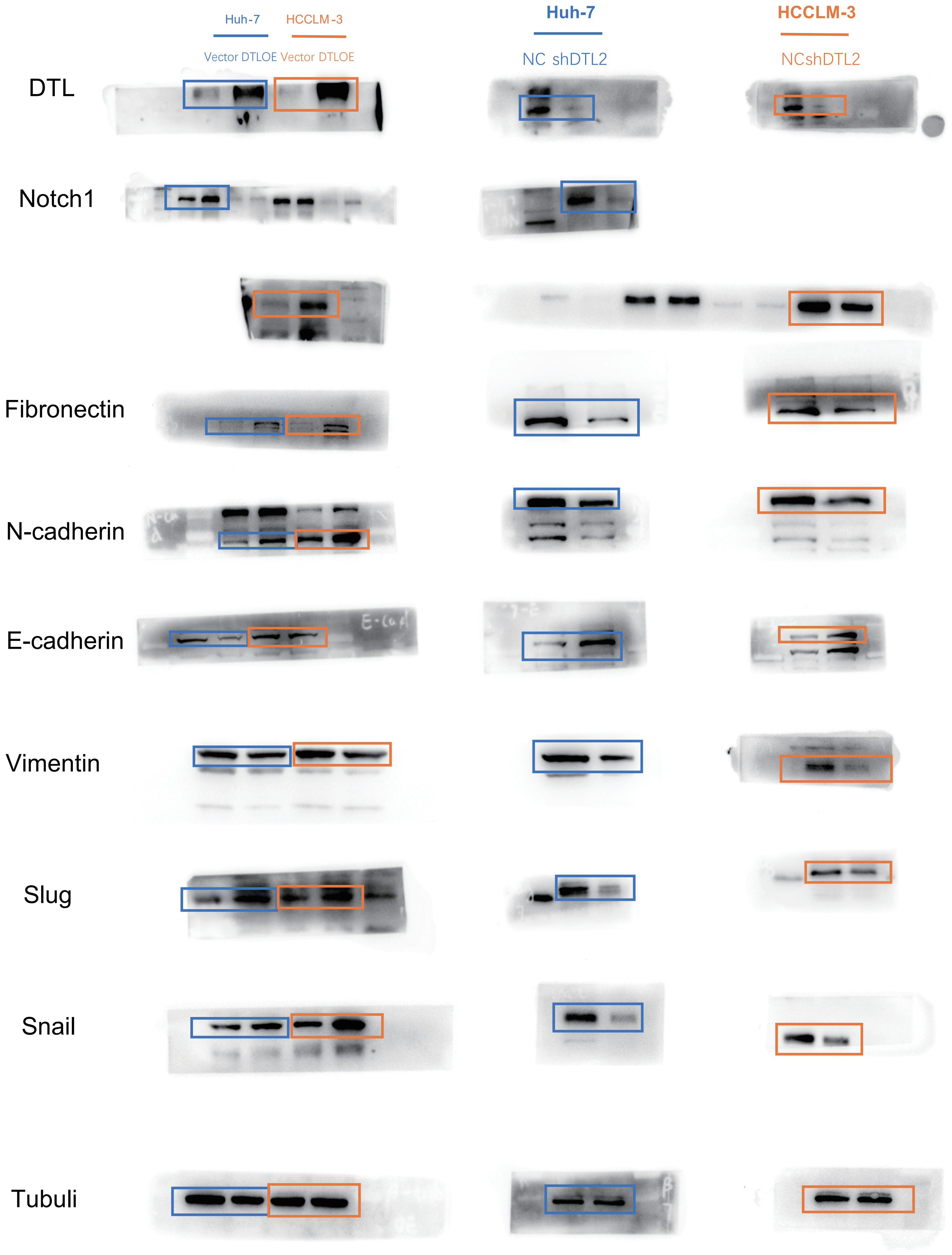

Full unedited blots for figure 7G

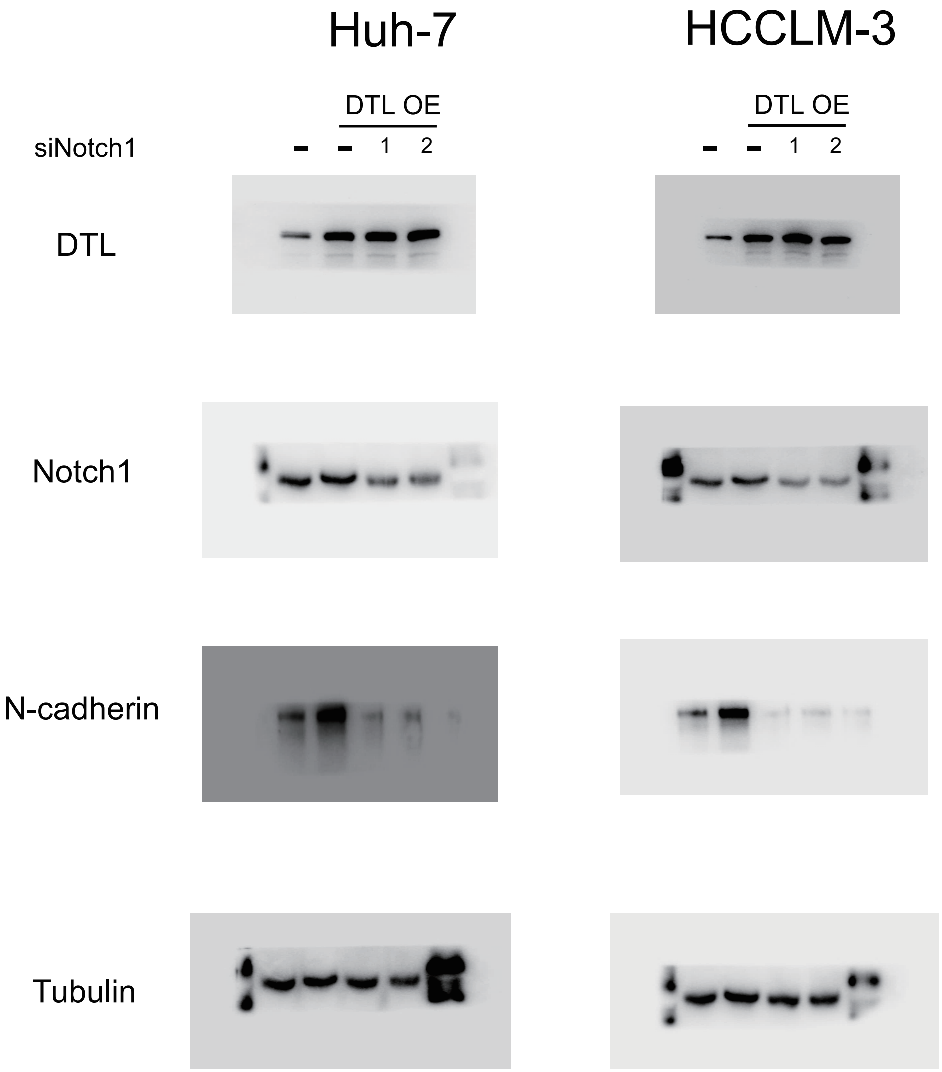

Full unedited blots for figure 8A-C

Fig 8B Fig 8A
IP IgG SLTM IP IgG DTL

IB DTL

IB SLTM

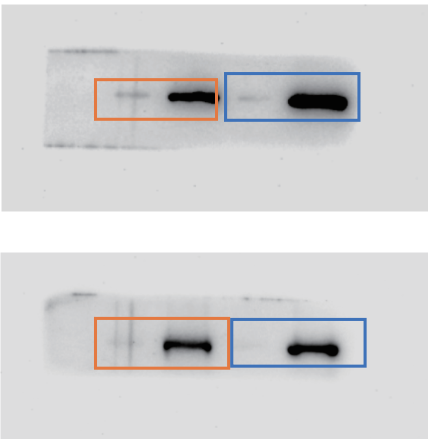

Input Fig 8A Fig 8B

IB DTL

IB GAPDH

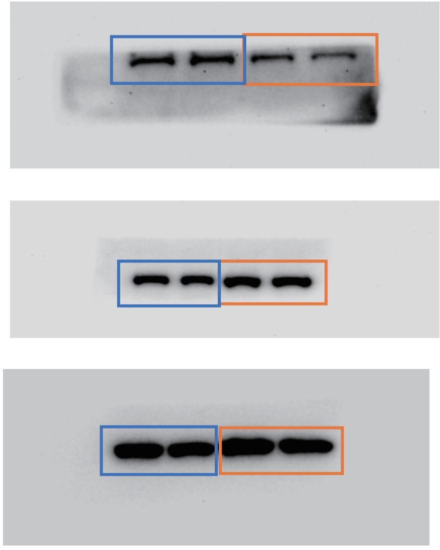

Fig 8C

IB Ubiquitin

IB Flag

IB HA

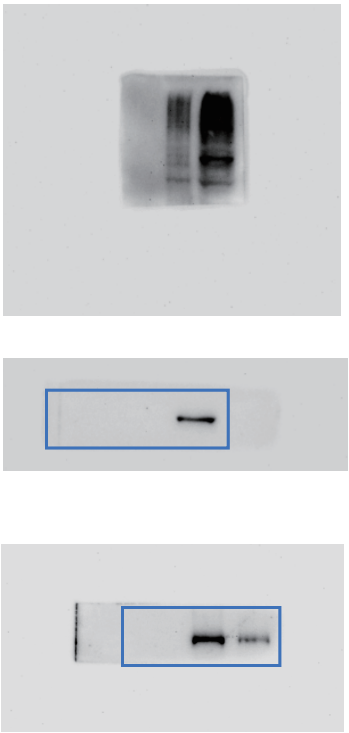

Full unedited blots for figure 8E-F

Fig 8E

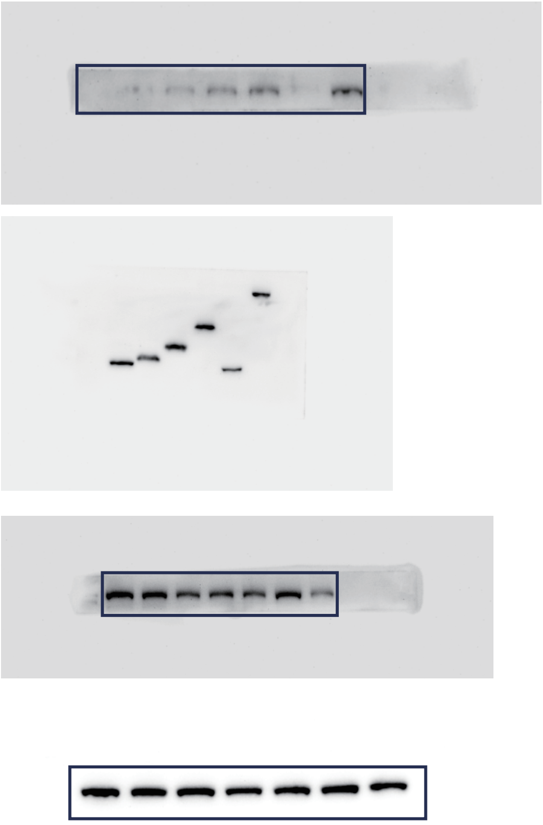

Fig 8F

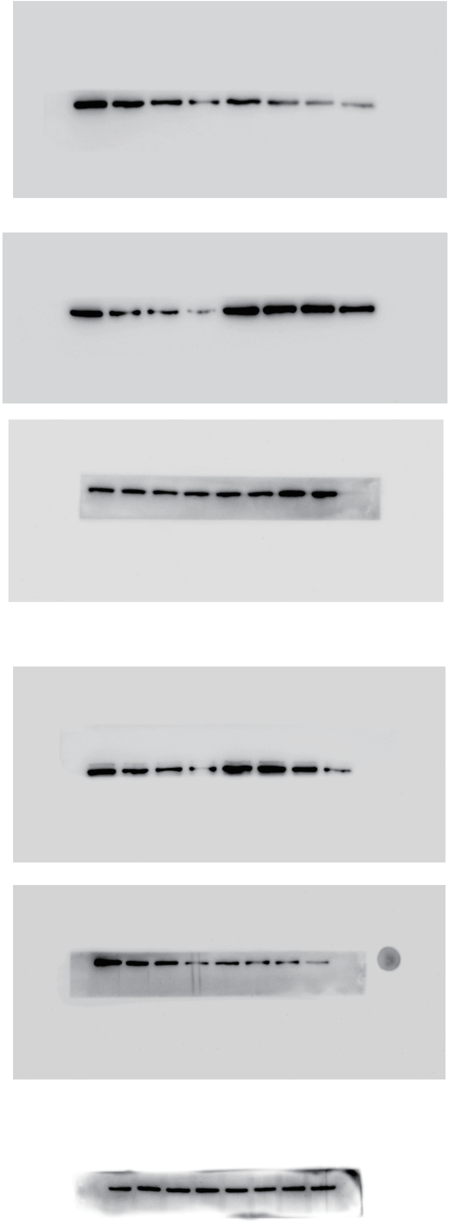

Full unedited blots for figure 8H

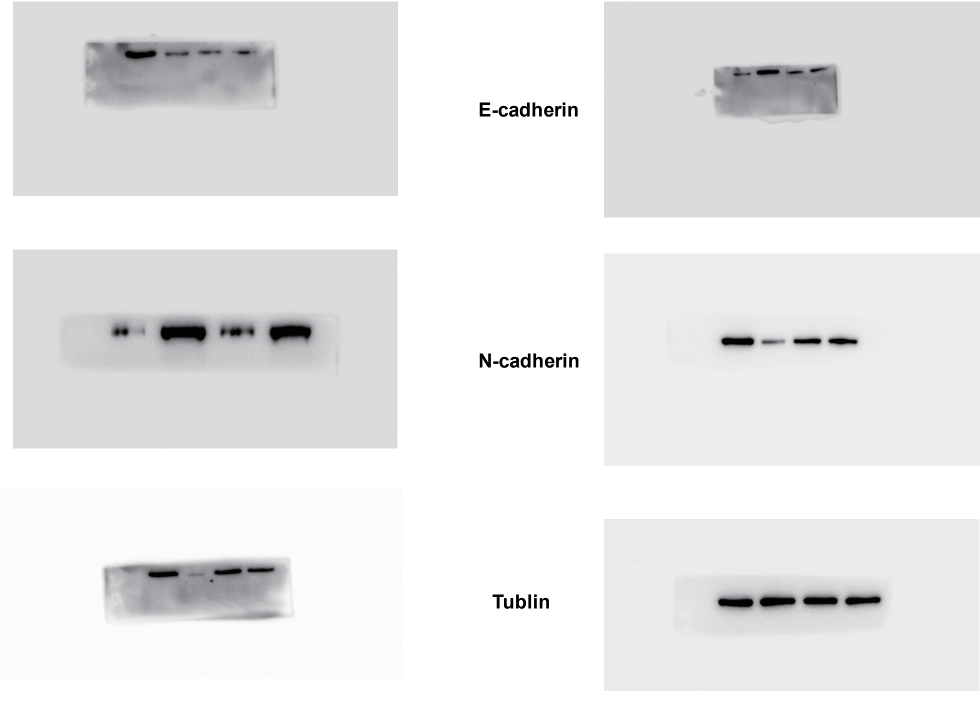

Full unedited blots for Supplementary Fig. 7A

Flag

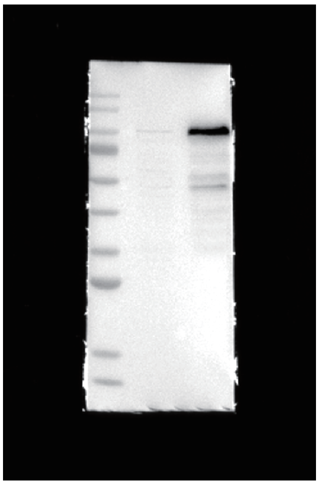

DTL

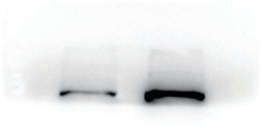

Tublin

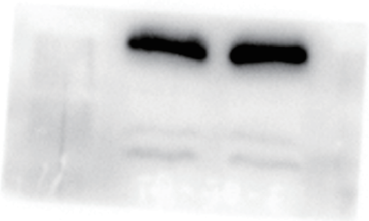

Supplement: Supplementary file 2 — Original Data [file 41419_2024_7089_MOESM2_ESM.pdf]
